# Fidelity, specialization, and evolution of *Paramecium* PolX DNA polymerases involved in programmed double-strand break DNA repair

**DOI:** 10.1093/nar/gkaf786

**Published:** 2025-08-19

**Authors:** Antonin Nourisson, Sophia Missoury, Soizick Lucas-Staat, Ahmed Haouz, Marc Delarue

**Affiliations:** Unit of Architecture and Dynamics of Biological Macromolecules, Université Paris Ci té, CNRS UMR 3528, 25-28 rue du Docteur Roux, Institut Pasteur, 75015 Paris, France; Sorbonne Université, Collège Doctoral, ED 515, 75005 Paris, France; Unit of Architecture and Dynamics of Biological Macromolecules, Université Paris Ci té, CNRS UMR 3528, 25-28 rue du Docteur Roux, Institut Pasteur, 75015 Paris, France; Unit of Architecture and Dynamics of Biological Macromolecules, Université Paris Ci té, CNRS UMR 3528, 25-28 rue du Docteur Roux, Institut Pasteur, 75015 Paris, France; Plate-forme de Cristallographie-C2RT, Institut Pasteur, Université Paris Cité, CNRS UMR 3528, 75015 Paris, France; Unit of Architecture and Dynamics of Biological Macromolecules, Université Paris Ci té, CNRS UMR 3528, 25-28 rue du Docteur Roux, Institut Pasteur, 75015 Paris, France

## Abstract

Repairing programmed DNA double-strand breaks (DSBs) is crucial in the lifecycle of *Paramecium tetraurelia*, especially during its sexual reproduction phase when its somatic polyploid macronucleus is lost. The formation of a new macronucleus involves programmed genome rearrangements, introducing DNA DSBs at ∼45 000 loci. *Paramecium tetraurelia* employs a non-homologous end joining (NHEJ) mechanism for the faithful repair of these DSBs. There are four genes encoding DNA polymerases of family X in the genome, one of which was found recently to colocalize with other NHEJ proteins in the nucleus. Here we have characterized all four enzymes and shown that they are generally very faithful. They fall into two functional classes that may specialize in the distinct repair contexts encountered during DSB DNA repair. Biochemical assays, site-directed mutagenesis, and X-ray structures of mutants of human Polλ incorporating sequence determinants from *P. tetraurelia* PolX or metazoan Polβ are used to investigate the origin of their fidelity. Our findings suggest that *Paramecium* PolX enzymes may represent evolutionary intermediates between metazoan Polβ and Polλ. A general classification of DNA PolXs based on clustering methods indicates that our results can be generalized to plant DNA PolXs (Polλ-like) involved in DSB DNA repair generated by CRISPR–Cas9 engineering.

## Graphical Abstract

**Graphical Abstract:**
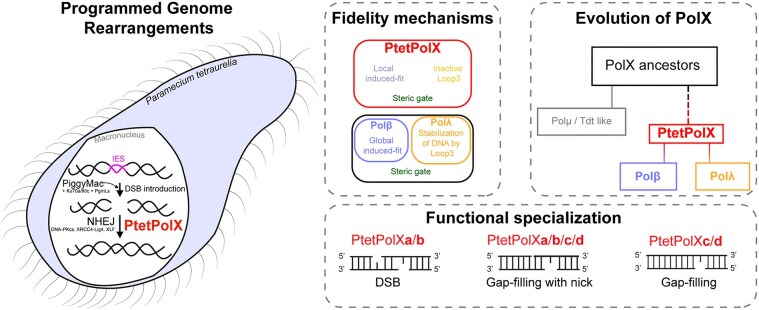

## Introduction

**Figure 1.**
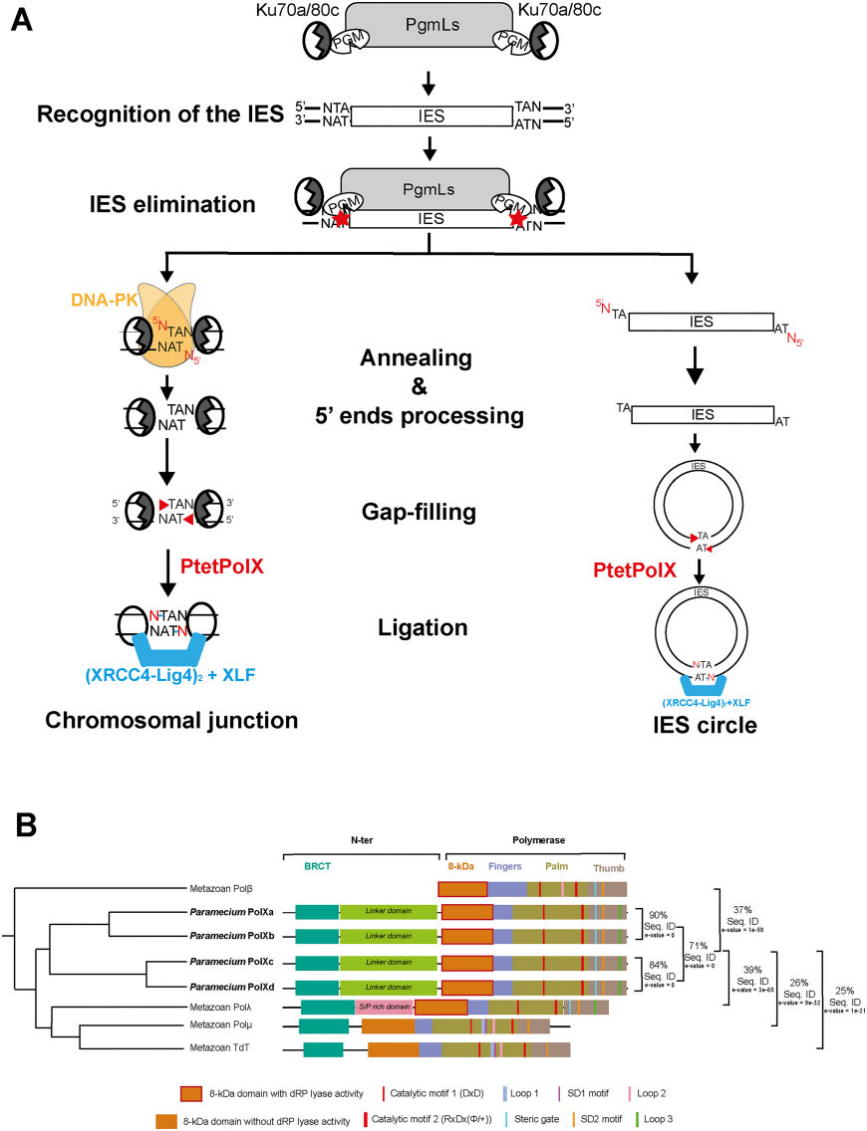
Role of PolXs in IES elimination during PGR in *P. tetraurelia*. (**A**) After IES elimination by Pgm and the PgmL proteins, the introduced DSBs are repaired by the NHEJ repair pathway, involving Ku70a/80c, DNA-PKcs, PolX, and XRCC4-Ligase IV. *Paramecium* PolXs (Ptet-PolXs) are involved in the gap-filling step, during which their role is to accurately synthesize the missing base on each strand. (**B**) *Paramecium* PolXs display a domain composition and sequence motifs similar to metazoan PolXs, especially the NHEJ-related Polλ and Polμ as well as V(D)J-related TdT. They share high sequence identity to one another and are divided into two groups (PolXab and PolXcd). Their closest homolog among metazoan PolXs is Polλ (39% of sequence identity), but they are also homologs of Polβ (37% of sequence identity), despite their additional BRCT domain. The domains are described under the figure, and the most important sequence motifs are indicated.

*Paramecium tetraurelia* exhibits nuclear dimorphism, with a single cell containing both a micronucleus (MIC), used for sexual reproduction, and a macronucleus (MAC), utilized for gene expression [1]. The MAC harbors up to 1600 copies of the 72 Mb somatic genome, while the diploid MIC genome (108 Mb) contains additional sequences, including internal eliminated sequences (IES) [2]. During the sexual reproduction cycle of *P. tetraurelia*, the MAC undergoes chromosome fragmentation, and a new MAC is formed from a copy of the MIC through extensive genome replication and rearrangements [3, 4]. These large-scale alterations, known as programmed genome rearrangements (PGR), entail the precise removal of ∼45 000 IESs. IESs are excised from the MAC DNA by a domesticated transposase known as PiggyMac (Pgm), which is thought to form active complexes with non-catalytic Pgm-like proteins [5, 6]. The DNA elimination complex induces double-strand breaks (DSB) specifically at dinucleotide 5′-TA-3′ sites flanking the IES, followed by the elimination of the 5′ terminal nucleotide (Fig. 1A). Recent studies revealed the participation of a specific Ku70/80 heterodimer in this complex [7] along with the Ligase IV complex [8–10], and presumably XLF, in a specialized NHEJ mechanism dealing with programmed DNA DSBs. While the NHEJ system is *a priori* capable of ensuring fidelity in coordinated DNA DSB repair, especially in the presence of cohesive ends [11, 12], the removal of the 5′ nucleotide at each DNA end following DSB induction necessitates an additional gap-filling step before ligation, involving at least one DNA polymerase to make the two DNA ends compatible (Fig. 1A). These findings prompt the question of the fidelity of the DNA polymerase involved [13, 14].

Four DNA PolX genes, namely *POLXa, b, c*, and *d*, can be identified in the genome of *P. tetraurelia* [15]. In addition, colocalization in the nucleus of one of them, PolXa, with the other proteins of NHEJ (Ku70 and Ligase 4) has just been described [15]. Here we will focus on these four PolXs in various DNA repair contexts and try to identify the origin of their high fidelity.

### State-of-the-art in structural studies and sequence analysis of other eukaryotic polX

In metazoan [16], the gap-filling step is performed by DNA polymerases of family X (PolX), such as Polλ [17] or Polμ [18]. Other members of the PolX family participate in short-patch base excision repair (BER) (Polβ [19, 20]) or V(D)J recombination (terminal deoxynucleotidyl transferase (TdT) [21]). Both Polλ and Polμ are capable of dealing with a wide spectrum of nucleic acid substrates resulting from different scenarios of DNA DSBs [22].

Apart from the N-terminal BRCT domain followed by a linker domain, PolX proteins consist of an 8-kDa domain and a DNA polymerase catalytic domain (Fig. 1B). The 8-kDa domain aids in recognizing the 5′ phosphate DNA ends and exhibits a deoxyribose phosphate (dRP) lyase activity in Polβ and Polλ [20, 23]. The DNA polymerase catalytic domain is composed of 3 subdomains (fingers, palm, and thumb) and contains the catalytic aspartate triad, a steric gate motif (YFTGS, implicated in discriminating NTPs from dNTPs), three loops (Loop1, Loop2, and Loop3), and two sequence determinant (SD1 and SD2) motifs (Fig. 1B). Loop1 and SD1 motif are specific to Polμ and TdT [24]; their role is to help in the stabilization of a 5′ recessing template strand upstream of the DSB, followed by a short microhomology (MH) region [25–27]. The SD2 motif sequence is unique to each group of PolX. In TdT, the SD2 motif has been proposed to bind an additional divalent Zn^2+^ ion, but its precise role remains unclear [28]. For TdT and Polμ point mutations in this SD2 motif deeply affect the polymerase activity [28, 29]. In Polλ, there are only five residues in Loop1 instead of 17–19 in Polμ, and they are involved in fidelity by controlling the placement of the primer DNA in the active site [30]. The role of Loop2 is not completely understood, as it is located far from the active site, but mutagenesis and functional studies show that it is important in Polβ, together with SD2 [31, 32]. Loop3 is unique to Polλ and related homologues; its function is not completely understood, but it has been observed by X-ray crystallography to undergo a large conformational change upon substrate fixation and catalysis in the active site [33]. In the presence of a correct incoming dNTP, Loop3 relocates closely to the DNA template strand and stabilizes the active site [33]. Conversely, with incorrect dNTPs, Loop3 appears to be flexible, making it challenging to see it in crystal structures. In these instances, it does not interact closely with DNA, which is sub-optimally positioned in the active site for catalysis. Altogether, the recent detailed kinetic crystallography experiments of Polλ described in [33] show that Loop3 likely plays a role in catalysis and potentially contributes to fidelity in Polλ in the case of a single gap to be filled, but this requires further experimental functional evidence. Loop3 is also probably involved when the gap to be filled is larger than one nucleotide [33], which requires scrunching of the extra template base [34].

Notably, PolXs enzymes involved in NHEJ typically share a permanently closed conformation [34] and display low fidelity, particularly Polμ and TdT [35]. On the other hand, the BER-associated Polβ displays the highest fidelity of metazoan PolX, which is mainly attributed to a large conformational change occurring when the right dNTP binds [36], named a typical induced-fit mechanism. In the presence of DNA and an incorrect incoming dNTP, the catalytic domain is in an open conformation with its thumb subdomain located away from DNA, and R258 forms a salt bridge with catalytic D192, diverting it from the active site and preventing catalysis [31]. However, when DNA and the correct dNTP are present in the active site, the enzyme adopts a closed form, in which the thumb subdomain closes and stabilizes DNA in the active site, and the side chain of F272 (involved in the steric gate) now separates R258 and D192. In this active conformation, R258 forms a new salt bridge with the glutamate residue of the SD2 motif (NEY), and D192 is now available for catalysis. [36]. Interestingly, in Polβ, the dNTP and DNA binding sites are not preformed, which is in contrast with the apo form of Polμ and Polλ, where there is a preformed DNA binding site, and with the apo form of Polλ that binds dNTPs in the absence of DNA [37]. The potential mutator effect of this prestabilization of dNTPs is counterbalanced by a hydrophobic cluster that regulates the transition to an active state of the catalytic site [37, 38].

### Presentation of the four *P. tetraaurelia* PolXs

The four *Paramecium* PolX genes, *POLXa*, *b*,*c*, and *d*, stem from two whole genome duplications [39] and form a common group. These genes are paralogues and exhibit varying degrees of sequence identity. At the protein sequence level, PolXa and PolXb share 90% sequence identity, while PolXc and PolXd share 84% of sequence identity, and the two subgroups collectively share 71% of sequence identity (Fig. 1B). Notably, messenger RNA (mRNA) levels of PolXa are upregulated during PGR in *P. tetraurelia* [40]. The four PolXs share significant sequence similarities with both human Polβ (37% sequence identity) and Polλ (39% sequence identity), mainly restricted to the polymerase domain, and they exhibit common features in their domain organization, including a putative N-terminal BRCT domain, a linker domain, an 8-kDa domain containing residues possibly involved in dRP lyase activity, and a catalytic DNA polymerase domain resembling both DNA polymerases λ and β (Fig. 1B). The BRCT domains of *P. tetraurelia* PolXs do not align well with the ones of metazoan PolXs by BlastP searches but AlphaFold confidently predicts the presence of a domain in all four variants that was attributed to a BRCT domain by InterPro, expasy ScanproSite, MyCLADE, and structural alignments with other PolX BRCT domains (with RMSD < 5Å) (Supplementary Fig. S1). This is true for all variants of PolXa, b, c, and d. However, the linker domain appears to be different from the one in Polλ, as it is highly SP-rich in Pol λ and predicted by PONDR to be disordered (confirmed by AlphaFold), while it is not in *P. tetraurelia* PolXs and could be partially ordered according to AlphaFold (Supplementary Fig. S1). The BRCT domain is responsible for the association of the PolX with the rest of the NHEJ complex, especially the Ku70/80 heterodimer. Recently, it was shown that the linker domain is responsible for the co-localization of PolXa, b with other components of the NHEJ machinery in the nucleus [15]. Here, we ask what makes the polymerase domain of these DNA PolX special with respect to human Polμ and Polλ catalytic domains and how they contribute to fidelity in *P. tetraurelia*’s NHEJ process, ensuring fully reliable repair after the excision of IES by Pgm [8, 12].

In the following, we present the functional characterization of Ptet-PolXs. We show that different Ptet-PolXs exhibit good fidelity on distinct physiological DNA substrates, while their kinetic parameters remain comparable to those of HsPolλ. Sequence analysis led us to explore two distinct fidelity mechanisms for Ptet-PolXs, one based on Polβ (presence of a positively charged residue next to the first catalytic aspartate) and the other on Polλ (presence of Loop3). Using biochemical assays, we tested the first one and demonstrated that indeed a specific lysine plays a crucial role in fidelity, akin to Polβ’s R258. The second mechanism, involving Loop3, was also investigated. Surprisingly, while we can confirm that Loop3 contributes to fidelity in Polλ, it does not play a similar role in Ptet-PolXs. Because we could not crystallize *P. tetraurelia* PolX itself with various DNA substrates, we set out to crystallize mutants of human Polλ incorporating sequence determinants from *P. tetraurelia* PolX or metazoan Polβ: these structures indicate that a Loop3-based fidelity mechanism is incompatible with the fidelity determinants of Polβ, which is based on a large conformational change (open-to-close transition). Altogether, our results suggest that Ptet-PolXs may represent evolutionary intermediates between metazoan Polβ and Polλ and that their features might serve as a model for plant Polλ-like DNA polymerases, recently studied in the context of DNA repair after Crispr-Casp9 modifications [41].

## Materials and methods

### Clustering of the PolX family

Six thousand five hundred PolX sequences were obtained by PSI-BLAST [42] of the NCBI [43] non-redundant database, using the sequence of Ptet-PolXa as a probe. Sequences were sorted by query coverage, and the last chosen sequence displayed the following parameters: query coverage= 52%; *e*-value = 2 × 10^−37^; % identity = 32.02%. To obtain representative sequences for the bacterial PolXs and yeast PolIV groups, three other PSI-BLASTs were performed, using as probes PolX from *Thermus thermophilus* [NCBI ID: WP_096410530.1], PolX from *Deinococcus radiodurans* [NCBI ID: WP_010887112.1], and PolIV from *Saccharomyces cerevisiae* [NCBI ID: AJP37443.1]. From each search, the top 250 sequences were chosen.

The FASTA file containing 7250 final sequences was processed by the CLANS web-utility from the MPI Bioinformatics Toolkit [44, 45]. The clustering simulation was conducted using the Java version of CLANS [46] with default parameters. The sequences were randomly distributed in 3D space, converging to individual clusters after several hundred steps of the simulation. The simulation was let to run for a total of 20 000 steps, during which no further modification of the positions appeared. The simulation was run without applying a *P*-value cut-off: additional simulations with cut-offs (10^−10^ and 10^−20^) resulted in a shrinking and fragmentation of the clusters, leading to the impossibility to observe known relationships between PolX. Clusters were curated manually. Several independent simulations converged to almost identical cluster distributions, with no discrepancy regarding the critical details. Simulations not enriched with bacterial or yeast PolX sequences resulted in equivalent distributions.

### Sequence multialignment

A multialignment of various eukaryotic PolXs sequences was computed using PSI-Coffee [47] and encompasses all regions of interest located in the DNA polymerase domain. Graphical representation of the multiple sequence alignments was generated using ESPript 3 [48], with secondary structures from the human Polλ (PDB 7M43) provided for reference.

### Domain prediction

The online version of PONDR [49] was used to predict potential disordered regions in *P. tetraurelia* and human PolXs. AlphaFold3 [50] was used to predict their structure. The PAGSTscore was calculated over a sliding window of 10 residues and smoothed over a window of 5 residues. The attribution of the domains was done by structural alignments with BRCT domains from other PolX (PDB 2JW5, 2DUN, 2COE, for HsPolλ, HsPolμ, and HsTdt, respectively), and by sequence annotation using InterPro [51], Expasy PROSITE [52], and MyCLADE [53–55].

### Wild-type and mutant constructs of PolX

In this study, we purified different versions of *Paramecium* PolXs. PolXd (ParameciumDB PTET.51.1.P1010039) and PolXb (ParameciumDB PTET.51.1.P0360066) were expressed and purified as a full-length construct (PolXdFL and PolXbFL), while four proteins lacking residues 1–266 (including the N-terminal BRCT domain and the linker domain) were generated: PolXaΔNter (WT sequence: ParameciumDB PTET.51.1.P0210235), PolXbΔNter, PolXcΔNter (WT sequence: *Paramecium*DB PTET.51.1.P0460033), and PolXdΔNter. Additionally, three mutant versions of PolXaΔNter were created: PolXaΔNter-K534A (mutation K534A), PolXaΔNter-K534R (mutation K534R), and PolXaΔNter-Loop3β, where residues 581 to 588 (Loop3) were replaced by the corresponding sequence of Pol β (GVA). A PolXdΔNter-Loop3β construct was also created the same way.

Wild-type (WT) versions of human Polλ (Uniprot Q9UGP5) and Polβ (Uniprot P06746) were also produced. Furthermore, five mutant versions of human Polλ were generated, all featuring the following mutations originally described in [33]: Δ1–241 (ΔNter), replacement of residues 464–472 (Loop1) with the equivalent sequence in Polβ (KGET), and C543A. The protein that carries these first 3 mutations will be referred to as Polλxt in the following. The specific mutations in the other constructs were as follows (Table 1): I492R (Polλxt-R), I492R, and residues 528 to 530 (SD2 region) were replaced by the corresponding—much shorter—Polβ sequence NEY (Polλxt-R-SD2β), a single mutation I492K (Polλxt-K), and two mutations—I492K and E529D (Polλxt-K-SD2X). Additionally, another construct based on human Polλ, named Polλ-loop3β, was produced and purified, with residues 539–547 (Loop3) replaced by the corresponding sequence of Polβ (GVA).

**Table 1.** Mutant constructs of human Polλ used for enzymatic and structural studies of the fidelity mechanisms of Ptet-PolXs

Mutant	Crystallization facilitating mutations	Specific mutations	Expected role of mutations
Polλxt	Δ1–241 [464–472] KGET C543A	None	Facilitate crystallization (Δ1–241 and [464–472] KGET) and diffraction (C544A)
Polλxt-R		I492R	Partly confer to Pol λ the Polβ’s induced fit mechanism (R258 of Polβ)
Polλxt-R-SD2β		I492R, [528–530] NEY	Confer to Pol λ the Polβ’s induced fit mechanism (R258 and SD2 motif of Polβ)
Polλxt-K		I492K	Partly confer to Pol λ the Ptet-PolXs putative induced fit mechanism (K534 of Ptet-PolXs)
Polλxt-K-SD2X		I492K, E529D	Confer to Pol λ the Ptet-PolXs putative induced fit mechanism (K534 and SD2 of Ptet-PolXs)
Polλ-loop3β	None	[539–547] GVA	Deletion of Loop3, replacement by the equivalent residues of human Polβ

All the codon-optimized sequences for WT constructs are indicated in Supplementary Table S1, and mutated constructs and used primers are described in Supplementary Table S2.

### Cloning, overexpression, and purification of *Paramecium* PolXs

The synthetic genes encoding Ptet-PolXs were optimized for expression in *Escherichia coli* and synthesized using Thermo Fisher’s GeneArt service. These genes were then cloned into a modified pRSF1-Duet expression vector with a cleavable N-terminal 14-histidine tag. For the PolXdFL and PolXbΔNter constructs, cloning was done using New England Biolabs and Anza (Thermo Fisher Scientific) restriction enzymes, while polymerase chain reaction (PCR) and NEBuilder HiFi DNA Assembly (New England Biolabs) were utilized for the other WT and ΔNter constructs. The mutant constructs of PolXaΔNter were generated via site-directed mutagenesis performed by PCR (primers are indicated in Supplementary Table S2) and using the KLD enzyme kit (New England Biolabs). The gene encoding PolXdΔNter-Loop3β was commercially synthesized by Genscript and inserted into the modified pRSF1-Duet expression vector with a cleavable N-terminal 14-histidine tag.

*Escherichia coli* BL21 Star (DE3) cells (Invitrogen) were transformed with the engineered plasmids and cultured at 37°C in LB medium with kanamycin resistance selection. Induction was carried out at OD = 0.6–1.0 with 1 mM IPTG, followed by overnight incubation at 20°C. After harvesting, cells were homogenized in buffer A [50 mM Tris–HCl, pH 8 (or Hepes pH 7 for PolXbFL and PolXcΔNter), 600 mM NaCl, 10 mM imidazole], sonicated, and centrifuged to remove bacterial debris. The resulting lysate supernatants were treated with Benzonase (Sigma–Aldrich) and protease inhibitors (Thermo Fisher Scientific) before purification.

The proteins of interest were isolated by purifying the clarified cell lysates on a HisTrap column, using buffer A [50 mM Tris–HCl, pH 8 (or Hepes pH 7), 600 mM NaCl, 10 mM imidazole] as the washing buffer and 500 mM imidazole in the elution buffer. For the PolXbΔNter construct, a different washing buffer (25 mM Sodium Phosphate, pH 8, 1 M NaCl) was utilized to avoid nucleic acid contamination. The collected proteins were then diluted to 75 mM NaCl and repurified on a HiTrap Heparin column with an elution at 1 M NaCl. Both purification columns were from Cytiva.

Protein purity was evaluated using sodium dodecyl sulfate polyacrylamide gel electrophoresis (SDS–PAGE) 4%–15% or 4%–12% gels with a molecular weight ladder (Precision Plus Protein, Biorad) as a control. The enzymes were concentrated using Amicon Ultra 30k or 10k MWCO centrifugal filters (Merck), flash frozen in liquid nitrogen, and stored directly at −20°C until further use.

### Cloning, overexpression, and purification of human Polβ

The gene encoding human Polβ was commercially synthesized by Genscript and inserted into the modified pRSF1-Duet expression vector with a cleavable N-terminal 14-histidine tag. Production and purification followed the same protocol as described for the previous constructs, with the use of specific buffers: Buffer A (50 mM Tris–HCl, pH 8, 500 mM NaCl, 10 mM imidazole), Buffer B (50 mM Tris–HCl, pH 8, 500 mM NaCl, 500 mM imidazole), Dilution buffer (50 mM Tris–HCl, pH 8), Buffer C (50 mM Tris–HCl, pH 8, 100 mM NaCl), and Buffer D (50 mM Tris–HCl, pH 8, 1 M NaCl).

### Cloning, overexpression, and purification of human Polλ and mutant constructs

The gene expressing WT human Polλ was commercially synthesized by Genscript and inserted into the modified pRSF1-Duet expression vector with a cleavable N-terminal 14-histidine tag. Production and purification followed the same protocol as described for the previous constructs, utilizing the following buffers: Buffer A (50 mM Tris–HCl, pH 8, 500 mM NaCl, 10 mM imidazole, 1 mM EDTA, 1 mM DTT, 5% glycerol), Buffer B (50 mM Tris–HCl pH 8, 500 mM NaCl, 500 mM imidazole, 1 mM EDTA, 1 mM DTT, 5% glycerol), Dilution buffer (50 mM Tris–HCl, pH 8, 1 mM EDTA, 1 mM DTT, 5% glycerol), Buffer C (50 mM Tris–HCl, pH 8, 100 mM NaCl, 1 mM EDTA, 1 mM DTT, 5% glycerol), and Buffer D (50 mM Tris–HCl, pH 8, 1 M NaCl, 1 mM EDTA, 1 mM DTT, 5% glycerol).

All mutant constructs were obtained by PCR (primers are indicated in Supplementary Table S2) using the KLD enzyme mix (New England Biolabs) and purified similarly to the WT protein. An additional step of size-exclusion chromatography was performed on a HiLoad Superdex 200 16/60 PG gel filtration column (Cytiva) in Storage Buffer (50 mM Tris–HCl, pH 8, 100 mM NaCl). All purified proteins were concentrated to 16 to 20 mg/mL and stored at −80°C after flash freezing in liquid nitrogen until further use.

### Gap-filling and DSB polymerase assays

Two DSB assays were performed, with different substrates, to reproduce *in vitro* the closest possible conditions to the physiological context of activity of Ptet-PolXs. One substrate was supposed to mimic the DNA junction between two IES after the IES elimination from the genome (“DSB IES junction”). The used oligonucleotides were designed to respect the loose consensus described by Klobutcher *et al.* [56] and the GC content of 15%–20% found in the IES [57] (here the GC content is 18.8%). Their sequences were the following: 5′-FAM-AAATATCTAACTATATATCACAACT-3′, 5′-p-TACAGTTATAATTCTTACTTTATAAAAG-3′, 5′-TACAGTTGTGATATATAGTTAGATATTT-3′, 5′-CTTTTATAAAGTAAGAATTATAACT-3′. The other substrate (“DSB chromosome junction”) was designed to mimic the chromosomal junction after the IES removal. That kind of junction does not display any consensus (even loose), but their GC content is slightly different, around 25%–30% [57], and was therefore reproduced in this *in vitro* assay with 28.3% of GC nucleotides. The oligonucleotide sequences were the following: 5′-FAM-TTCCTCAAATAGTAAGATT-3′, 5′-p-TAGATATTTGCCATTAACAG-3′, 5′-TACAATCTTACTATTTGAGGAA-3′, 5′-CTGTTAATGGCAAATAT-3′. These assays were conducted in a buffer comprising 50 mM Tris–HCl (pH 8.4), 5 mM MgCl2, 100 mM NaCl, 0.1 mM EDTA, 5 mM DTT, 10% glycerol, and 0.1 mg/mL bovine serum albumin. Each reaction mixture contained 1 μM of hybridized DNA and 250 μM of each dNTP. Reactions were incubated for 50 min at 27°C (the optimal growth temperature of *P. tetraurelia*) with various concentrations of Ptet-PolXs.

Two gap-filling assays were conducted, with different substrates. One was named “gap-filling junction,” with a continuous template strand, using the following oligonucleotides: 5′-FAM-AATCACCAGTACGCCGTTGCGT-3′, 5′-p-TATCGCCATGACGCGGTTCTGGTCC-3′, 5′-GGACCAGAACCGCGTCATGGCGATACACGCAACGGCGTACTGGTGATT-3′. The other was named “gap-filling junction with nick on template strand,” composed of the following oligonucleotides: 5′-FAM-AAATATCTAACTATATATCACAACT-3′, 5′-p-TACAGTTATAATTCTTACTTTATAAAAG-3′, 5′-TACAGTTGTGATATATAGTTAGATATTT-3′, 5′-CTTTTATAAAGTAAGAATTATAACTG-3′. Assays performed using these substrates were conducted in a reaction buffer consisting of 50 mM Tris–HCl, pH 7.5, 50 mM KCl, 5 mM MgCl_2_, 1 mM DTT, and 5% glycerol. Reaction solutions comprised 100 nM of all oligos and either 1 mM dGTP or a mixture of all four dNTPs (1 mM each). Reactions were incubated for 30 min at 27°C with 1 nM of polymerase (Ptet-PolXs) or for 5 min at 37°C with 50 nM of polymerase (HsPolλ).

For all assays, prior to adding the protein, DNA was hybridized by heating up to 95°C and gradually cooled down to room temperature.

For the primer extension assay, the used oligonucleotides were the following: 5′-FAM-AATTGTCATAAGCTTATGCG-3′, 5′-p-TATCGCCATGACGCGGTTCTGGTCC-3′, 5′-GGGGTAGCTGCGCATAAGCTTATGACAATT-3′. Reactions were incubated for 30 min at 27°C with 1 μM of polymerase (Ptet-PolXs) or at 37°C with 50 nM of polymerase (HsPolλ).

All reactions were terminated by adding two volumes of a buffer containing 10 mM EDTA, 98% formamide, and 1 mg/mL bromophenol blue and stored at −20°C. Products were preheated at 95°C for 10 min, separated using denaturing urea-polyacrylamide (18%) gel electrophoresis, and visualized by FAM fluorescence on a Typhoon FLA 9000 imager. All oligonucleotides were obtained from Eurogentec, Eurofins, or Biomers; dNTPs from Fermentas (Thermo Fisher Scientific); and chemicals from Sigma–Aldrich.

### dRP lyase assay

For the dRP lyase assay, DNA was prepared using the following procedure: a 31-mer DNA strand (5′-GTACCCGGGGATCCGTACAGCGCATCAGCTGCAG-3′) and its complementary U-containing DNA strand (5′-CTGCAGCTGATGCGCUGTACGGATCCCCGGGTAC-3′), each at a concentration of 50 μM, were hybridized as described earlier. Next, four picomoles of hybridized DNA were mixed with USER3 mix (New England Biolabs) in ThermoPol buffer [20 mM Tris–HCl, pH 8.8, 10 mM (NH_4_)_2_SO_4_, 10 mM KCl, 2 mM MgSO_4_, 0.1% Triton^®^ X-100], and the mixture was incubated for 2 h at 65°C.

Subsequently, in a reaction volume of 10 μL, 1 μM of USER3-treated DNA was mixed with a 250 μM dNTP mix, 400 units of T4 DNA ligase, and 50 nM of each DNA polymerase (HsPolβ or PolXaΔNter) in an activity buffer (50 mM Tris–HCl, pH 7.5, 5 mM MgCl_2_, 50 mM KCl, 1 mM DTT, 1 mM ATP). The mixture was then incubated for 30 min at 27°C (for PolXaΔNter) or 37°C (for HsPolβ) and analyzed by urea-PAGE in denaturing conditions as described previously.

### Enzymatic steady-state characterization

DNA polymerization assays were conducted between 3 and 12 times using the methodology described earlier. Each assay utilized 1 μM of gap-filling DNA and 5 nM of DNA polymerases, with varying concentrations of dNTPs, and proceeded for 10 min at 27°C.

The resulting gels were subjected to analysis using ImageJ software, and quantification of the product DNA was carried out using Microsoft Excel. Enzyme velocity (measured in nM/min) was plotted against the concentration of dGTP. These plotted data points were then fitted to a nonlinear regression curve utilizing the Michaelis–Menten equation with GraphPad Prism 10 software. From the fitted curves, values for k_obs_ and K_M_ were obtained.

### Crystallization

For crystallization of Polλxt and its variants, the DNA substrate was prepared in the following way: an 11-mer template oligonucleotide (5′- CGGCAGTACTG-3′) was annealed with a 6-mer upstream primer oligonucleotide (5′-CAGTAC-3′) and a 5′-phosphorylated downstream 4-mer primer oligonucleotide (5′-pGCCG-3′) in a 1:1:1 ratio. The crystallization plates were prepared according to the procedure outlined by Jamsen *et al.* [33]. In brief, the protein (16 mg/mL) was combined with DNA in a 1:2 molar ratio and incubated at 4°C for 2 h. Following the addition of 2 mM dTTP and 10 mM CaCl_2_, the mixture was further incubated for 2  h on ice. Crystallization drops were set up at 4°C by mixing in a 1:1 ratio the Polλ-DNA-dNTP ternary complexes with a crystallization solution consisting of 20 mM Bicine, pH 7.5, 300 mM Na-K tartrate, and 14%–20% PEG 600/1000/10K/20K/Smear Low/Smear Medium/Smear High. The resulting crystals were flash-frozen in liquid nitrogen after being cryoprotected with the crystallization solution supplemented with 25% ethylene glycol.

### Data collection and refinement

X-ray crystallographic data were collected at the SOLEIL synchrotron (St. Aubin, France), utilizing beamlines PX1 and PX2A. The data were processed using XDS [58] with the XDSME pipeline or autoPROC [59]. The strong diffraction anisotropy of λSD2β dataset was taken into account with StarANISO (Staraniso GlobalPhasing, https://staraniso.globalphasing.org). Subsequently, the structures were solved by PHASER (https://www.globalphasing.com/) molecular replacement using an HsPolλ model (PDB ID: 7M43) as template and refined in Buster [60], with manual reconstruction performed using Coot [61]. Details for all datasets are summarized in Table 2.

**Table 2.** Crystallographic statistics for datasets of crystals of DNA polymerase λ mutants with dNTP insertion site occupied opposite A in the presence of Ca^2+^

	Polλxt-R-SD2β	Polλxt-K-SD2X
Data collection		
Space group	H32	P 21 21 21
a. b. c (Å)	149.90 149.90 272.15	56.4 62.47 139.57
*α*. *β*. *γ* (°)	90 90 120	90 90 90
Wavelength (Å)	0.9801	0.9801
Resolution (Å)	117.17 - 3.54 (4.02 - 3.54)	46.55 - 2.12 (2.18 - 2.12)
Estimated resolution limit (Å)^a^	5.49 5.49 3.34	
R-pim	0.66 (1.19)	0.04 (0.69)
Completeness (%)	91.70 (74.2)	99.80 (97.5)
Multiplicity	17.70 (15.2)	13.40 (13.7)
I/σ(I)	7.80 (1.80)	16.60 (1.6)
CC1/2	80.5% (74.7%)	99.9% (79.4%)
Refinement		
No. of reflections	5991	28 561
R_work_ / Rfree	0.243 / 0.284	0.218 / 0.255
No. non H atoms		
Macromolecules	5811	2953
Ligands	0	33
Solvent	24	313
Protein geometry		
RMSD—bonds (Å)	0.006	0.008
RMSD—angles (°)	0.79	0.83
Ramachandran favored (%)^b^	96.17	97.8
Ramachandran outliers (%)^b^	0.32	0.00
Poor rotamers (%)^b^	5.01	1.89
Clashscore^b^	5.49	3.45
B-factors(Å²)		
Mean B-factor	72.45	54.82
*Macromolecules*	72.73	53.94
Ligands	0	104.42
Solvent	6.7	57.84

Data in the highest resolution shell are shown in the parenthesis.

^a^Values calculated after truncation by STARANISO. Estimated resolution limits along the three crystallographic directions of the reciprocal lattice a*, b*, c*.

^b^Values obtained with MolProbity.

## Results

### Comparison of Ptet-PolXs sequences with other DNA PolX

**Figure 2.**
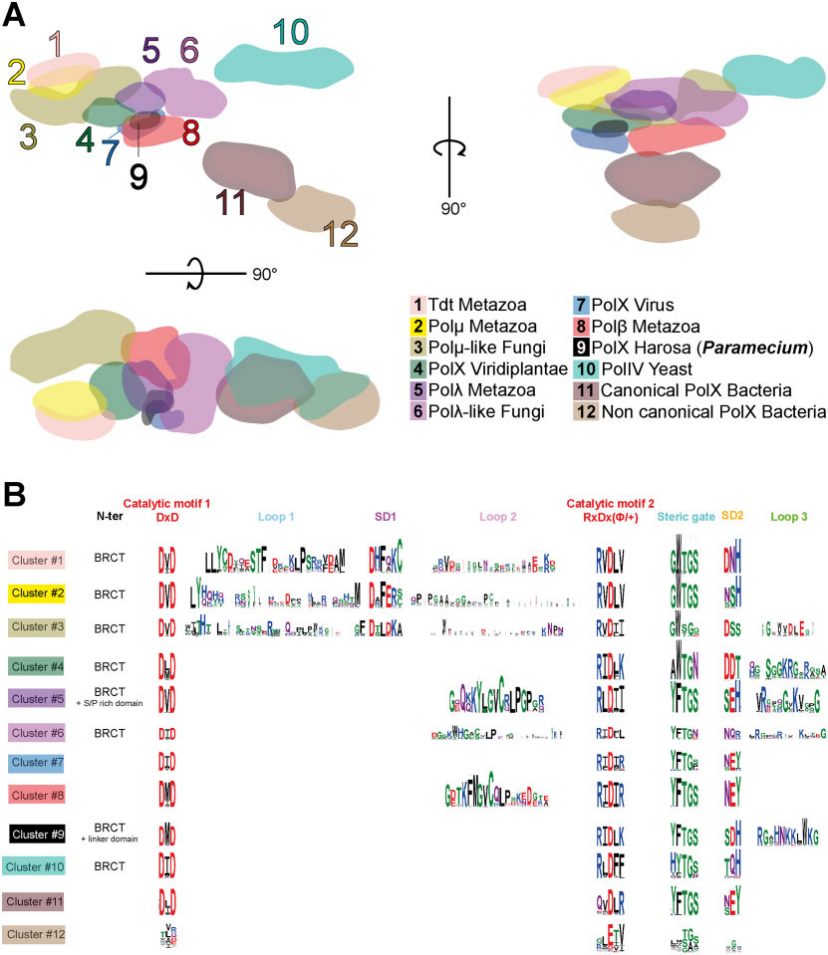
Cluster analysis of PolXs sequences along with the sequence determinants and logos of each group. (**A**) Non-hierarchical clustering of PolXs sequences. A 3D distribution of 7250 PolXs sequences was generated with CLANS [47] using the pairwise BlastP scores of sequence similarity between individual sequences. Three planar projections in different directions of this distribution are shown. The 12 clusters of PolXs sequences are colored and numbered. Their names are given on the bottom right. Ptet-PolXs are in the Harosa group (#9), also called SAR (Stramenopiles, Alveolatas, Rhizaria) or TSAR (if Telonemia are included). (**B**) Sequence motifs of the 12 PolX clusters. Each cluster is represented by a sequence logo, where the height of each residue reflects its conservation. The N-terminal region is treated separately and indicates the presence of additional domains (BRCT, S/P-rich region, or linker), without conservation analysis.

To understand the position of Ptet-PolXs in the PolX family and determine the possibility of shared fidelity mechanisms with known PolXs, we used the CLANS classification method [46] on 7250 representative PolX sequences from diverse organisms (*Paramecium*, metazoan, fungi, viridiplantae, and bacteria) obtained by PSI-BLAST. This method is based on the Fruchterman–Reingold algorithm: it randomly distributes the sequences as dots in a 3D space, and then it uses pseudoforces derived from all-against-all BlastP searches to regroup similar sequences. It was previously used to study the classification of family B DNA polymerases [62], AEP superfamily [63], and to reclassify the DNA polymerase A family [64]. As indicated in Fig. 2A, 12 clusters were identified manually after convergence of the refinement process using 20 000 cycles. We confirmed the accuracy of the method by finding the same relationships between the different subgroups of PolXs that were known and analyzed previously using a smaller dataset [65]. Figure 2A shows that the metazoan clusters Polμ (#2) and TdT (#1) are close, as expected; the same is true for the Polλ (#5) and Polβ (#8) clusters. Also, other clusters are positioned close to Polλ, such as PolXs from plants (cluster #4) that were classified as Polλ until now [16, 66] or fungal PolXs (clusters #3 and #6) [67]. The most divergent PolXs clusters are those including yeast Pol IV (or Pol4) and bacterial PolXs. Interestingly, bacterial PolXs are separated into two clusters, containing canonical and non-canonical PolXs, as described by Prostova *et al.* [68].

We then compared representative sequence motifs of PolXs from each cluster (catalytic motifs, SD1/2 motifs, loops 1/2/3, and steric gate motifs). The obtained logos (Fig. 2B) allowed us to compare the 12 groups and define specific motifs that can be used to determine the group of any new PolX. Those results, along with sequence alignments with known eukaryotic PolXs (Supplementary Fig. S2), indicated that Ptet-PolXs and the other sequences from their group (cluster #9, associated with the clade Harosa that includes ciliates) share similarities with both Polλ and Polβ, including the steric gate and residues involved in dRP lyase activity. They are distant from TdT and Polμ, as indicated by a short Loop1 and a different SD1 motif [69].

Ptet-PolXs also exhibit a similarity with Polβ in their second catalytic motif (RIDLK). In metazoan Polβ, this motif comprises five conserved residues: RIDIR. The C-terminal arginine residue (R258) plays a crucial role in the induced-fit mechanism of Polβ described in the introduction [31] and is involved in the high fidelity of the enzyme through an exchange of salt-bridge partners upon binding the correct incoming dNTP. Unusually among PolXs, *Paramecium* PolXs display a positively charged residue at this position (K534). They also have an equivalent for all the other residues involved in this induced-fit mechanism: the three catalytic aspartates, the steric gate F506, and a negatively charged residue at the second position of the SD2 motif (NEY in Polβ, SDH in Ptet-PolX). On the basis of this conservation of equivalent residues (especially the positively charged side chain in the second catalytic motif), we formed the hypothesis that Ptet-PolXs could also benefit from an induced-fit mechanism to achieve a good fidelity. In the following, we will test this hypothesis experimentally.

Additionally, Ptet-PolXs display a Loop3 sequence, like metazoan Polλs and related sequences (plant PolXs and Polλ-like PolXs of fungi) that is more positively charged than in metazoan Polλs (pI of 10.3 versus 8.23 in HsPolλ). Since Loop3 is involved in DNA stabilization during correct catalysis in human Polλ [33], it could have the same role in Ptet-PolXs. This hypothesis will also be tested here by site-directed mutagenesis.

### Characterization of Ptet-PolXs enzymatic activities, kinetics, and specificity

**Figure 3.**
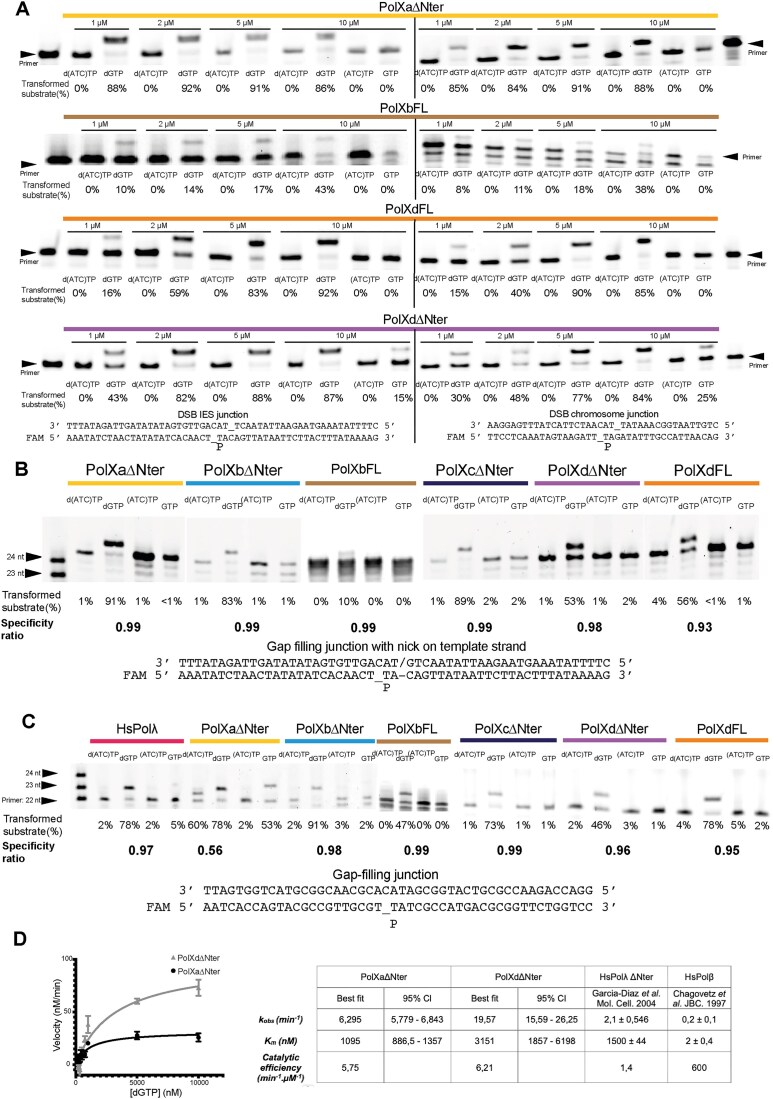
Characterization of Ptet-PolXs enzymatic gap-filling activities in different DNA DSB or SSB contexts. (**A**) PolXaΔNter, PolXbFL, PolXdFL, and PolXdΔNter are active *in vitro* on the two physiological substrates produced by IES excision, in a concentration-dependent manner and with excellent specificity (dGTP versus all the three other dNTPs). PolXaΔNter achieves almost 90% of transformation into the +1 product already at around 1 μM, while PolXd achieves it in the 2–5 μM range. PolXdΔNter is the only one that incorporates some (15%–25%) rGTP at high concentration (10 μM), and PolXbFL is less active than the three others, possibly due to a lower solubility of the enzyme. (**B**) *Paramecium* PolXs exhibit gap-filling activity on a secondary physiological substrate after the first incorporation on one strand but before ligation. They efficiently discriminate against NTPs and incorporate dGTP with high fidelity. In the indicated DNA sequence, underscores represent gaps, dashes indicate phosphodiester bonds, and slashes denote the absence of a phosphodiester bond between two nucleotides. (**C**) *Paramecium* PolXs display gap-filling activity similar to hPolλ when using a continuous template strand. All enzymes, except PolXaΔNter, exhibit good fidelity and selectivity by incorporating only dGTP. PolXaΔNter shows lower specificity and can also incorporate GTP. (**D**) Steady-state kinetics characterization of *Paramecium* PolX. PolXaΔNter and PolXdΔNter have different kinetics profiles in gap-filling, but display a similar catalytic efficiency, which surpasses HsPolλ’s. Their behavior in gap-filling is more similar to HsPolλ than to HsPolβ. Activities of PolXaΔNter (*n* = 12) and PolXdΔNter (*n* = 3) were assessed with increasing concentrations of dGTP, employing a gap-filling substrate. Left panel: Velocity curves for PolXa and PolXd as a function of dGTP concentration. The error bars correspond to the standard deviation on the measurements. Right panel: Kinetic values derived from the plots for each DNA polymerase. The optimal fit values and the 95% confidence intervals (CI) are provided. Literature data for HsPolλ and HsPolβ under analogous conditions are included.

To characterize the enzymatic activity of Ptet-PolXs, we tested their activity on various DNA substrates (see the “Gap-filling and DSB polymerase assays” section in “Material and methods”) (Fig. 3). Two physiological substrates (DSB IES or chromosome junctions) were initially used to mimic the context in which Ptet-PolXs operate: DSB intermediates generated during IES elimination, either at chromosomal junctions (Fig. 1A, right panel in Fig. 3A) or at IES–IES junctions forming circular intermediates (Fig. 1A, left panel in Fig. 3A). These substrates contain the conserved TA dinucleotide and present a DSB, with the template nucleotide positioned in *cis* to the extending strand, as in the gap-filling step of PGR (Fig. 1A). They also recapitulate *in vivo* specific features such as GC content and consensus sequences. In the absence of the 2-bp microhomology between the upstream and downstream DNA strands, they resemble short primer extension scenarios, which Ptet-PolXs can perform, albeit inefficiently (Supplementary Fig. S4A). In these assays, all tested Ptet-PolXs exhibit concentration-dependent activity on both substrates (Fig. 3A) but PolXaΔNter already reached full transformation into the +1 product at 1 μM. All polymerases incorporated only the correct incoming nucleotide. PolXaΔNter, PolXbFL, and PolXdFL efficiently discriminate between dNTPs and NTPs, as they do not incorporate ribonucleotides, even at high concentrations. However, PolXdΔNter incorporates some GTP (25%), which is unexpected given the conservation of Polλ’s steric gate residues (YFTGS, Fig. 2B and Supplementary Fig. S2). The difference in behavior between the full-length and truncated versions of PolXd suggests that the BRCT and/or linker domain may play a role in ribonucleotide discrimination in this Ptet-PolX. Furthermore, the distinct behavior of PolXaΔNter (similar to PolXbFL) suggests that this requirement does not apply to PolXa when encountering these substrates, indicating a possible functional specialization of PolXa in processing these substrates during PGR, consistent with its known overexpression pattern. The low activity of PolXbFL is surprising but this observation should, however, be taken with some caution, as purification of this protein systematically yielded low amounts and displayed poor solubility, in contrast to other PolX homologs. PolXaFL could not be purified at all.

Following the first dNTP incorporation on these substrates, two possible pathways can occur: (i) DNA ligase IV seals the filled DNA strand, allowing a gap-filling step followed by another ligation step and (ii) ligation does not occur yet, leading to gap filling in the presence of a nick in the template strand, followed by a ligation of both strands. We tested the activity of the four Ptet-PolXs in these two conditions.

In the “gap filling junction with a nick in the template strand” condition (Fig. 3B), all constructs exhibited robust and selective activity, incorporating the correct dNTP into the substrate. PolXaΔNter, PolXbΔNter, and PolXcΔNter displayed the highest apparent activity, transforming ∼90% of the substrate with the correct incoming dNTP, as indicated by their specificity ratio (SR = [Int(+1) with dGTP]/[Int(+1) with mix of incorrect dNTPs] + [Int(+1) with dGTP]) that exceeds 0.9. In contrast, PolXbFL and PolXd(ΔNter or FL) converted only 10% to 50% of the substrate, albeit with the correct dNTP (SR > 0.9). Importantly, in this context, all constructs did not incorporate ribonucleotides, including PolXdΔNter, in contrast to its behavior in the previous assays.

In the last physiological condition tested (Fig. 3C, “gap filling junction”), the same Ptet-PolXs constructs were compared to human Polλ. PolXbFL, PolXbΔNter, PolXcΔNter, PolXdFL, and PolXdΔNter displayed similar activity to previous assays, maintaining good specificity and not incorporating NTPs, comparable to HsPolλ. However, as in the previous assays, the two constructs of PolXb exhibited a pyrophosphorolysis activity, as evidenced by its ability to remove nucleotides from the initial substrate. The most notable difference from previous results was that PolXaΔNter efficiently incorporated GTP (50%) and showed a reduced specificity ratio (0.56), whereas all other tested constructs maintained a specificity ratio above 0.95. These results suggest that PolXc and PolXd may be specifically adapted for such contexts in *Paramecium*.

Altogether, all PolXs have an excellent specificity ratio. The use of different DNA DSB substrates indicates that PolXa is possibly the first one recruited on stage in the DNA repair process. PolXc and PolXd may be involved in the later stages of the DNA repair, after the first ligation step has occurred, hence in a less crucial stage.

We evaluated the dRP lyase activity of the PolXaΔNter construct, considering that its closest metazoan homologues, Polβ and Polλ, both exhibit this activity *in vitro*, and that the four Ptet-PolXs all carry the residues involved in this enzymatic activity (Supplementary Fig. S2). In an *in vitro* assay designed to reconstitute a short-range BER context, PolXaΔNter indeed exhibits a dRP lyase activity comparable to HsPolβ (Supplementary Fig. S4B), which is likely shared by the three other Ptet-PolXs, as they also carry the needed residues.

**Figure 4.**
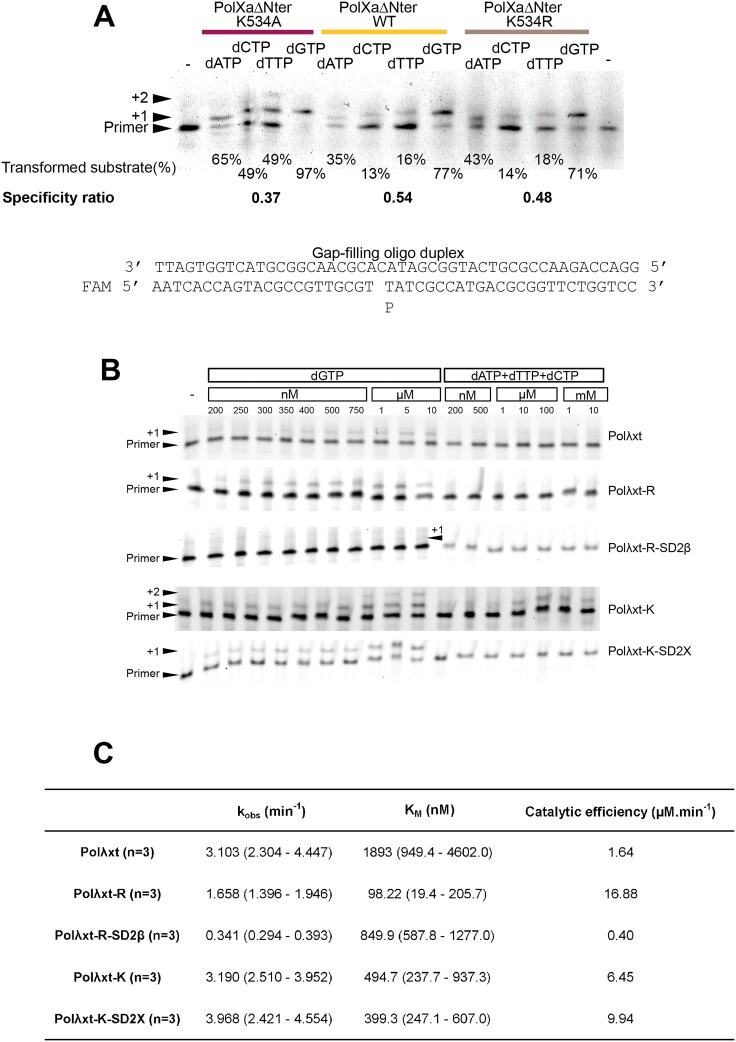
Activity of mutants for the test of a possible induced-fit mechanism for the fidelity of Ptet-PolXs carried out in part by K534. (**A**) Role of K534 in fidelity for Ptet-PolX. Single time-point, single-turnover fidelity assay was conducted with WT or mutant versions of *Paramecium* PolXaΔBRCT. The K534A construct displays a strong error-prone behavior, but the K534R mutation has a lower effect on fidelity. (**B**) Gap-filling assays were performed with WT (Polλxt) and different mutant versions of human Polλ, with increasing concentrations of correct and incorrect nucleotides. (**C**): Kinetic constants of dGTP insertion by the different mutant versions of Polλ. The value in parentheses stands for standard deviation estimated from *n* = 3.

To further compare their activity to human Polλ and β, we characterized the kinetics of incorporation of one representative from each subgroup (PolXa/b and PolXc/d), PolXaΔNter and PolXdΔNter, under the same conditions as those used previously for human Polλ [70] and Polβ [71], within a gap-filling context. As depicted in Fig. 3D, PolXaΔNter and PolXdΔNter exhibit distinct catalytic characteristics in this assay: the measured k_obs_ of PolXaΔNter is three times lower than PolXdΔNter but it has a three times higher affinity for dGTP, indicated by a lower K_M_. Ultimately, they demonstrate similar catalytic efficiencies of ∼6 min^−1^μM^−1^. Comparing these results with previous data reported for human Polλ and Polβ [70, 71] and data obtained with the Polλxt mutant (see Fig. 4C), their kinetic parameters more closely resemble those of Polλ than Polβ. Specifically, they exhibit a similar affinity for dGTP as HsPolλΔNter, with a K_M_ in the millimolar range, but also possess a 3–10 times higher turnover number. Consequently, they appear to be six times more efficient than HsPolλΔNter, but they display a low catalytic efficiency compared to HsPolβ.

### Mutants of Ptet-PolXs and human Polλ designed to probe a Polβ-like induced-fit mechanism

Considering the observed fidelity of Ptet-PolXs, we explored the first hypothesis for a potential molecular mechanism suggested by sequence analysis, involving a Polβ-like induced-fit mechanism. We first conducted an assay to compare the fidelity of WT and mutant versions of PolXaΔNter (Fig. 4A), focusing on K534, the equivalent of Polβ’s R258, which plays a central role in its induced-fit mechanism. The PolXaΔNter K534A protein exhibits strong misincorporation, incorporating any dNTP with a specificity ratio of 0.37 (compared to 0.54 for the WT enzyme). This mutation also affects enzyme kinetics, as this mutant is the only construct tested that converted almost 100% of the initial substrate with dGTP. By contrast, the K534R mutation has a much weaker effect on specificity and catalysis.

To further probe this mechanism, we engineered and purified five mutants of human DNA polymerase λ, the closest relative of Ptet-PolX, targeting residues that contribute to the induced-fit mechanism of Polβ or that are suspected to contribute to a similar mechanism of Ptet-PolXs. These constructs also included the following mutations to facilitate crystallization and diffraction [33]: Δ1–241, [464–472] KGET, and C543A. The reference construct (Polλxt) in this section carried all these three mutations. A full description of these mutants and their rationale is provided in Table 1.

The Polλxt-R construct contains the I492R mutation that involves the position equivalent to Polβ’s R258 residue, and the Polλxt-R-SD2β mutant has an additional mutation giving it the SD2 motif of Polβ (NEY). These mutants aim to determine whether introducing Polβ’s key induced-fit elements into Polλ enhances its catalytic efficiency and possibly its fidelity, as well as whether structural changes such as thumb domain closure and active-site rearrangement occur, as in Polβ.

Additionally, two mutants were generated to incorporate Ptet-PolXs-like features into Polλ: Polλxt-K (I492K) and Polλxt-K-SD2X (which also carries E529D, mimicking the SD2 motif of Ptet-PolXs, SDH). These mutants were designed to assess whether Polλ’s enzymatic behavior and structure would be affected similarly to Ptet-PolXs.

**Figure 5.**
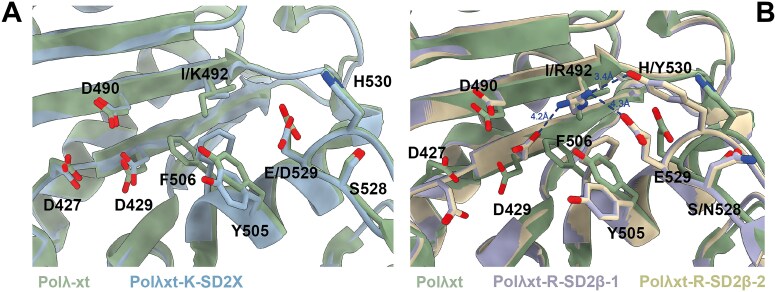
Comparison of the structures of the active site of two mutant versions of Polλxt and with Polλxt (PDB 7m43, in green). The residues of interest are highlighted, including catalytic residues (D490, D427, and D429), steric gate residues (Y271 and F272), SD2 motif residues (528, 529, and 530), and residue 492 in catalytic motif 2. Distances between residues are indicated in blue. In the structure of the Polλxt-K-SD2X mutant (**A**), K492 is flexible and cannot be entirely seen in the structure (Cβ and Cγ only), but all the catalytic residues are in place for catalysis. The Polλxt-R-SD2β mutant (**B**) displays two molecules in the asymmetric unit, which are superimposed here and named Polλxt-R-SD2β-1 and Polλxt-R-SD2β-2. In these structures, D429 is displaced from the catalytic site by R492, which is located between this catalytic aspartate and the SD2 motif, at a distance of ∼3.5–4 Å from both, possibly forming salt bridges with D429, E529, and Y530.

**Figure 6.**
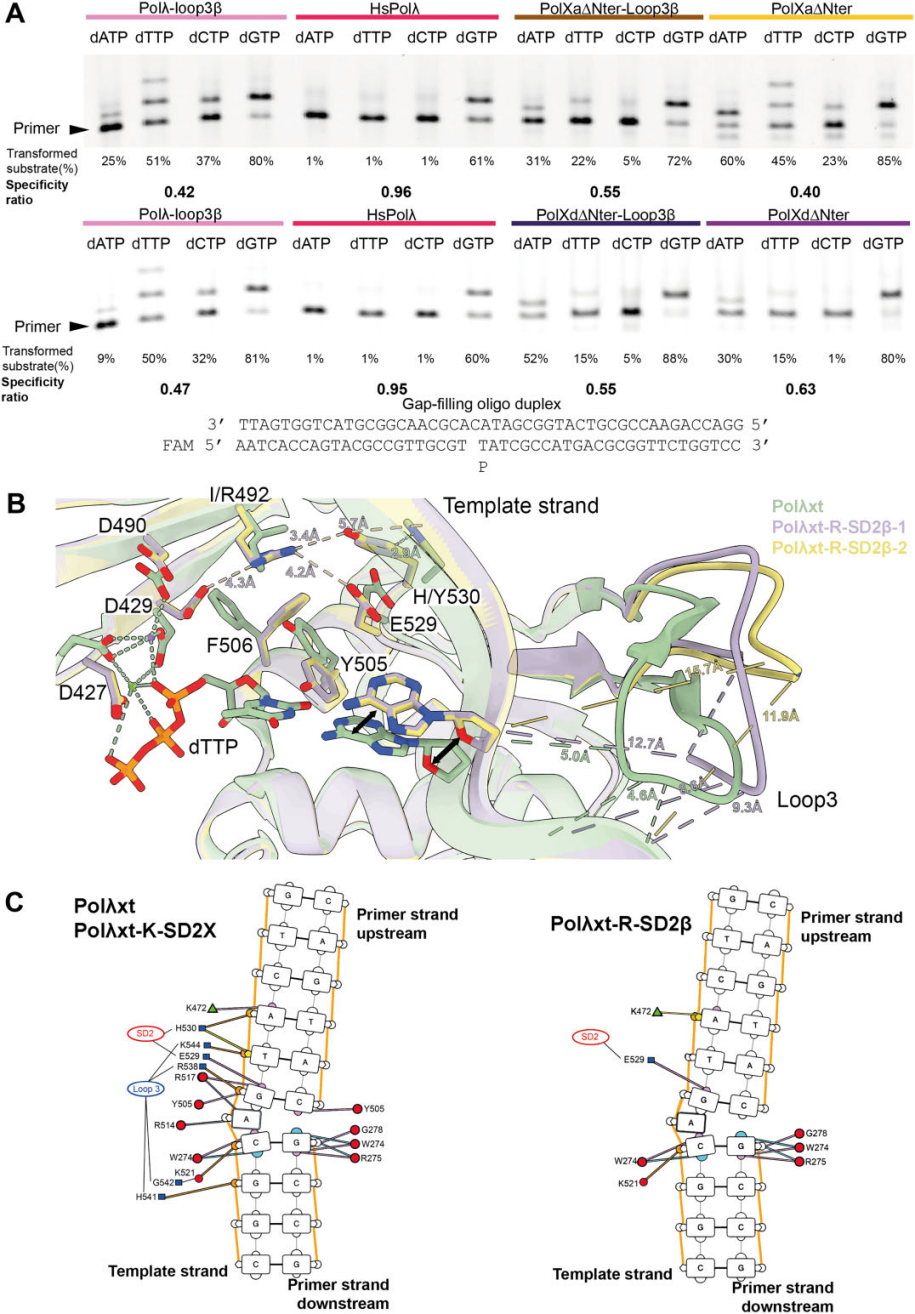
Role of Loop3 in catalysis and fidelity in Ptet-PolXs and human Polλ, and its interaction with the template DNA strand. (**A**) Role of Loop3 in fidelity for human Polλ and Paramecium PolXs. Single time-point, single-turnover fidelity assays were performed using a gap-filling DNA substrate with human Polλ, PolXaΔNter, and PolXdΔNter, either with or without their respective Loop3. In human Polλ, Loop3 deletion drastically reduces fidelity. In PolXaΔNter, Loop3 deletion appears to slightly increase fidelity, while it has no significant effect in PolXdΔNter. (**B**) Comparison of Loop3 interaction with the template DNA strand in one of the X-ray structures in a gap-filling context with a continuous template strand. In Polλxt (PDB 7m43, in green), Loop3 interacts with the DNA template (5 Å), and H530 is positioned to form a salt bridge with the DNA backbone. In the Polλxt-R-SD2β structure (shown in light purple and yellow), Loop3 is displaced (≥10 Å) and does not interact with the DNA. In the same structure, the mutated residue Y530 pushes the DNA phosphodiester backbone (>5 Å), leading to a displacement of the template strand and an improper positioning of the templating nucleotide for catalysis (bold arrows). (**C**) Comparison of DNA interactions in Polλxt and Polλxt-K-SD2X (left) versus the Polλxt-R-SD2β structures (right). In Polλxt and Polλxt-K-SD2X, several residues from the SD2 motif and Loop3 interact with the DNA, while most of these contacts are absent in the Polλxt-R-SD2β structures. Residues in red circles are located in α-helices, those in green triangles in β-strands, and those in blue squares in loops. The type of DNA interaction is color-coded: cyan for the minor groove, pink for the major groove, yellow for sugar moieties, gray for bases, and orange for phosphate groups.

After purification, all mutants underwent steady-state characterization in gap-filling context (Fig. 4B and C). The Polλxt-R-SD2β and Polλxt-K-SD2X mutants were further analyzed via X-ray crystallography (Fig. 5) in the presence of Ca^2+^ ions to prevent catalysis [72]. The Polλxt-R mutant exhibited reduced enzymatic activity compared to Polλxt, with a 2-fold lower k_obs_ and a 19-fold decrease in K_M_, indicating increased affinity for incoming nucleotides but decreased activity_._ When this mutant was further engineered to include the SD2 motif of Polβ (NEY), enzymatic activity was nearly abolished (10-fold lower k_obs_), despite an almost normal K_M_ in the millimolar range. Crystallographic data for this mutant produced different results compared to the other mutants. While the other constructs (including Polλxt obtained by Jamsen *et al.* [33]) crystallized in the P2_1_2_1_2_1_ space group with one molecule per asymmetric unit, this specific mutant crystallized in the H32 space group with two molecules in the asymmetric unit, displaying highly anisotropic diffraction data (Table 2), resulting in two very similar structures (see Fig. 5B) that differ only in their respective Loop3 (see Fig. 6B and Supplementary Fig. S5C and D). Both molecules exhibit a closed conformation, an absence of incoming nucleotide in the active site, and R492 positioned between catalytic D429 and the NEY SD2 motif (∼3.5–4 Å apart), suggesting stabilization via salt bridges. In these structures, Loop3 fails to engage with the DNA template strand (see Fig. 6B and C), which is improperly positioned for catalysis, with missing density for four and two residues in the two molecules of the asymmetric unit (see Supplementary Fig. S5C and D).

The Polλxt-K mutant, designed to mimic the putative Polβ-like induced-fit mechanism of *Paramecium* PolX, exhibited kinetic behavior similar to λref, particularly in k_obs_, but with a 3.8-fold lower K_M_, suggesting increased nucleotide affinity (Fig. 4C). The most striking feature of this mutant is its reduced fidelity: under steady-state conditions for correct dGTP incorporation, Polλxt-K was the only mutant to display clear misincorporations and double incorporations in the 1-nt gap-filling substrate (Fig. 4B).

The Polλxt-K-SD2X mutant, incorporating all residues potentially implicated in an induced-fit mechanism in *Paramecium* PolX, differs from Polλxt-K only by the E529D mutation, which mimics the SD2 motif of *Paramecium* PolX. Unlike Polλxt-K, it does not exhibit misincorporation, but it displays kinetics similar to Polλxt-K with slightly improved k_obs_ and marginally lower K_M_, leading to enhanced catalytic efficiency (1.64 μM^−1^ min^−1^ for Polλxt versus 9.94 μM^−1^min^−1^ for the Polλxt-K-SD2X mutant and 6.45 μM^−1^ min^−1^ for Polλxt-K). Structural analysis shows the flexibility of the K492 side chain, for which only the electron density of the β and γ methylene groups can be seen (see Supplementary Fig. S5A). However, no major conformational rearrangements are observed, and the polymerase domain remains in a closed conformation. This structure could be an equivalent of the active form of DNA polymerase β, displaying a flexible lysine instead of a clear attraction of the arginine by the SD2 motif.

In conclusion, these structural and functional studies show that K534 in Ptet-PolXs definitely contributes to enzyme specificity but requires additional residues such as those in the SD2 motif for full effect. Structurally, the mechanism does not fully resemble that of Polβ, as K534 appears flexible rather than stabilized. In any case, this process occurs without the large-scale domain rearrangements characteristic of Polβ.

### Determination of the role of Loop3 in fidelity

To assess the impact of the conserved Loop3 on the fidelity of Ptet-PolXs and HsPolλ, we generated and purified mutant versions of PolXaΔNter, PolXdΔNter, and HsPolλ, all lacking their respective Loop3 sequences, which are replaced by the one observed in Pol β: PolXaΔNter-Loop3β, PolXdΔNter-Loop3β, and Polλ-Loop3β. An assay was performed to compare the mutants with their WT counterparts (Fig. 6A). The results revealed distinct roles for Loop3 across the constructs. Specifically, the HsPolλ construct exhibited minimal errors, with a specificity ratio exceeding 0.9, while the Polλ-Loop3β mutant incorporated dATP, dTTP, and dCTP erroneously (specificity ratio between 0.4 and 0.5) and transformed 80% of the initial substrate, compared to 60% for the WT. In contrast, for the Ptet-PolXs constructs, the removal of Loop3 had little impact on fidelity. The specificity of PolXaΔNter was even slightly higher for the truncated version, while PolXdΔNter showed a slight decrease in specificity. However, the changes in specificity for the Ptet-PolXs mutants were minor when compared to the significant effect observed with HsPolλ.

Structural analysis provides a possible explanation for the enzymatic behavior of the Polλ-Loop3β mutant. In the Polλxt-R-SD2β mutant, Loop3 appears flexible, as indicated by the lack of density for four and two residues in the two molecules of the asymmetric unit, respectively (Supplementary Fig. S5C and D). It is positioned further from the DNA (peptide backbone 9–15 Å away, Fig. 6B), with high B-factors (83 and 89 Å², Supplementary Fig. S5E), in contrast to the Polλxt-R-SD2X and Polλxt structures, where Loop3 is closer to the DNA (peptide backbone 5 Å away, Fig. 6B), and DNA has lower B-factors (43 Å², Supplementary Fig. S5E). Upon Loop3 closure, salt bridges form between its positively charged residues (K544, R538, H541) and DNA phosphate groups. G542 also stabilizes a phosphate group by interacting with K521 (Fig. 6C). Other residues, including Y505, R514, R517, and R536, contribute to these interactions. H530 from the SD2 motif interacts with the phosphate group of the −3 nucleotide (Fig. 6C). In the Polλxt-R-SD2β mutant, where H530 is replaced with tyrosine, this interaction is lost. The tyrosine hydroxyl group pushes the DNA phosphate away, displacing the DNA and disrupting interactions. Only seven residues remain interacting with the DNA, insufficient for stabilization, causing the templating nucleotide to be mispositioned and preventing proper stabilization of the incoming nucleotide.

## Discussion

### *Paramecium* PolXs activity and kinetics resemble that of human Polλ

We assessed the activity of Ptet-PolXs with their physiological substrates encountered during PGR in *P. tetraurelia*. Our results indicate that Ptet-PolXs exhibit a concentration-dependent DNA polymerase activity in the context of DSB repair, with PolXa appearing to be the most specialized and efficient one among the four at the first step of DSB repair. It displays a high specificity, independently of its BRCT and linker domains, unlike PolXb or PolXd, which suggests a more defined role in this repair process. This apparent functional distinction between PolXa and PolXb is surprising, as they are closely related in sequence and are assumed to act redundantly *in cellula*. However, we note that PolXb was obtained with lower yields and solubility during purification, which may impact its apparent catalytic efficiency *in vitro*.

**Figure 7.**
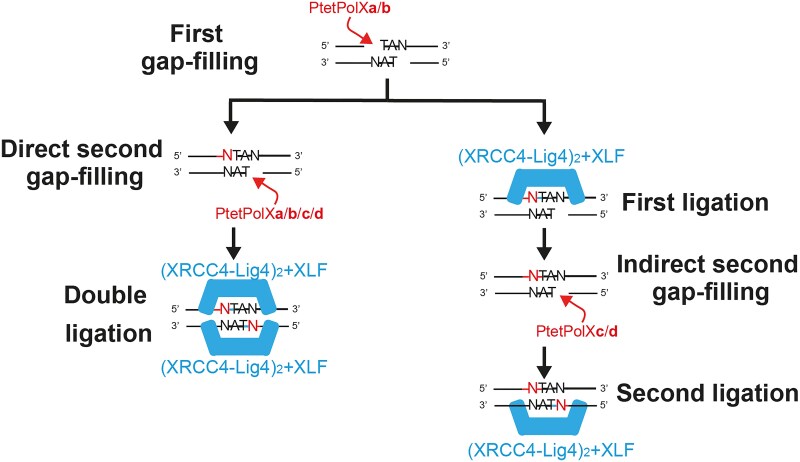
Proposed mechanism for sequential gap filling and ligation during PGR in *P. tetraurelia*. Following Pgm-induced DSBs, PolXa is required for the initial repair step and is strongly overexpressed, possibly assisted by PolXb, which is also upregulated. Two pathways may then operate. In the first (right branch), a ligation step precedes the second gap filling by PolXc or PolXd, and the last ligation step follows. In the second (left branch), a second gap-filling step is carried out by any of the four Ptet-PolXs, followed by two sequential ligation steps, one per strand. It is the first ligation step that is crucial: once it is carried out, it is not necessary anymore to have the NHEJ (short-range) complex in place to hold together the two DNA ends, as the dangers of illegitimate ligation have virtually vanished. The second ligation can occur later, without Ku70/80 or XLF. This model reflects a possible functional specialization among the four Ptet-PolX paralogs, where PolXa is the first DNA polymerase on site.

We further tested Ptet-PolXs in two subsequent gap-filling contexts: one with a nick on the template strand and one where the template strand remains continuous. When the template strand is nicked, all four Ptet-PolXs maintain good specificity, with various efficiencies. However, when the template strand is continuous, PolXc and PolXd appear to be the most efficient enzymes. This suggests a functional specialization among Ptet-PolXs during PGR (Fig. 7): following the induction of DSBs by Pgm, PolXa is essential for the initial repair synthesis and is accordingly upregulated. PolXb, also slightly overexpressed, may act redundantly or in support. Two alternative pathways can then be envisioned. In the first, an initial ligation event is followed by gap filling, specifically mediated by PolXc or PolXd; then a second ligation step can occur. In the second, a second round of gap filling is carried out by any of the four Ptet-PolXs, prior to two sequential ligation steps, one on each strand. This branching reflects a division of labor among the paralogs, possibly shaped by functional specialization.

Other results obtained in gap-filling context indicate an enzymatic resemblance between these PolXs and both human DNA polymerases β and λ, as they all exhibit gap-filling and dRP lyase activities, and do not incorporate NTPs. Their steady-state characterization, compared with published results obtained for human Polλ [70] and Polβ [71], provided more insights into their enzymatic mechanism and points to a higher similarity to Polλ. Thus, it is plausible that Ptet-PolXs employ a structural mechanism akin to that of Polλ and other NHEJ-related PolXs, maintaining a closed form of their active site throughout catalysis, contrary to Polβ.

To investigate the origins of their fidelity, we used a classification of PolXs into 12 subgroups that highlights their distinctive sequence features and their evolutionary relationships. A close examination of conserved sequences and key motifs led us to propose two distinct hypotheses that could account for Ptet-PolXs’ fidelity: a local induced-fit mechanism, as in Polβ, and a conformational change involving Loop3, as in Polλ.

### *Paramecium* PolXs fidelity can be explained by a possible induced-fit mechanism like in Polβ, but more local and without closing the whole polymerase domain

By testing the effects of point mutations in the second catalytic motif of Ptet-PolXs, we demonstrated that the presence of a positively charged residue at position 5 of this motif is essential for the enzyme’s fidelity. Enzyme kinetics and crystallographic structures of Polλ mutants provided further insights into this fidelity mechanism. Indirect structural evidence, combined with direct enzymatic characterization, suggests that Ptet-PolXs likely rely on a *local* induced-fit mechanism. This mechanism involves K534, potentially forming a salt bridge with one of the catalytic aspartates and residues from the SD2 motif. Although such a salt bridge is not directly observed in the structure, its potential role is supported by the observed effects of the mutations. Indeed, the lysine alone cannot ensure good fidelity, as its introduction into Polλ (Polλxt-K mutant) did not display good specificity. Instead, additional mutations in the SD2 motif were required, indicating that the SD2 residues are also essential for maintaining the enzyme’s fidelity.

### The differential role of Loop3 in the fidelity of Polλ and Ptet-PolXs

**Figure 8.**
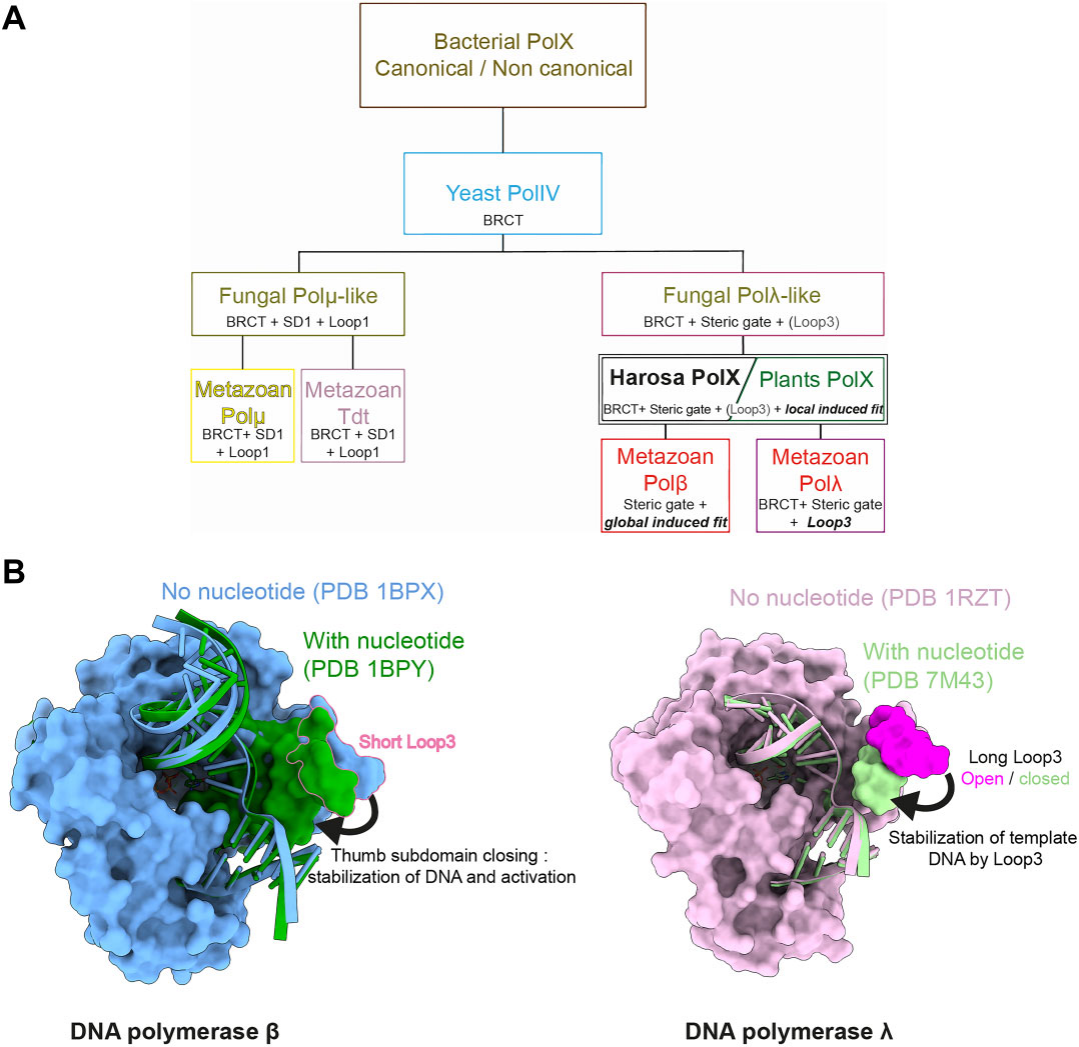
Evolution of structural specificities and fidelity mechanisms in the PolX family. (**A**) Putative evolutionary tree of all PolXs as inferred from the presence or absence of key sequence motifs and characteristic loops. Loops and mechanisms involved in fidelity are highlighted in bold italic. The presence of an inactive Loop3 in Harosa and Plants PolX is denoted by its inclusion in parentheses. In this model Polβ would have evolved by the loss of the BRCT domain and the acquisition of a larger (more global) induced-fit mechanism, while Polλ would have evolved by a fully functional Loop3 fidelity mechanism. (B) Comparison of conformational changes in the thumb subdomain between Polβ and Polλ. In Polβ, binding of the correct incoming nucleotide induces a global conformational change, where the entire thumb subdomain undergoes a large movement to close the polymerase domain and stabilize the DNA. In Polλ, the active site remains permanently closed. Upon correct nucleotide binding, only Loop3 (located at the tip of the thumb subdomain) moves to stabilize the DNA, while the overall conformation of the thumb subdomain remains unchanged.

Assays on mutants of HsPolλ and PolXaΔNter lacking Loop3 revealed distinct roles for Loop3 in Polλ and Ptet-PolXs in the context of a gap-filling activity with a continuous template strand (on which the kinetic crystallography experiments were done). In Ptet-PolXs, removal of Loop3 had minimal impact on enzyme activity and fidelity. In contrast, in Polλ, the deletion of Loop3 led to a significant increase in misincorporation, indicating that Loop3 plays a fundamental role in maintaining fidelity, likely through a DNA stabilization function (Fig. 6). We propose that this stabilization mechanism may involve an interaction between DNA and H530, the third residue of the SD2 motif in Polλ. Indeed, the interaction of H530 with DNA could be facilitated by the Watson–Crick base-pairing of the correct incoming nucleotide with the templating nucleotide in the active site. In Polλ, it has been shown that this correct hybridization triggers repositioning of the template DNA [73]. This suggests that, upon entry of the correct incoming nucleotide into the active site, its hybridization with the templating nucleotide is further stabilized by R514 and R517, which repositions the DNA and brings it closer to H530. This may enable their interaction and initiate a cascading interaction between DNA and Loop3 residues, “closing it” around DNA. Loop3 provides further stabilization of the DNA, promoting catalysis. Previous studies on Polλ have demonstrated that R517 stabilizes and controls the nascent base pair during dNTP-induced template strand repositioning [73, 74]: when R517 is mutated, DNA fails to reposition, and Loop3 is unable to undergo its conformational change. This proposed mechanism aligns with the model put forward by Showalter and Tsai [75], which suggests that nucleotide binding in the active site of polymerases triggers conformational changes, allowing the active site to close around the substrate, with fidelity being dictated by the energy difference between correct Watson–Crick base-paired and mismatched nucleotide incorporations. In Polλ, while the polymerase domain does not undergo full closure, the movement of Loop3 mimics the closing motion of the thumb subdomain in Polβ, leading to similar outcomes: stabilization of DNA in the active site and enhanced fidelity (Fig. 8B).

### *Paramecium* PolXs contain traces of fidelity mechanisms of both Polλ and Polβ

The lack of enzymatic activity in the Polλxt-R-SD2β construct suggests that the fidelity mechanisms of Polλ, based on Loop3, and of Polβ, relying on global induced fit and remodeling of the active site, cannot coexist in the same sequence and structural context. This incompatibility likely arises from Polλ’s evolution toward a permanently closed form, for which fidelity depends on a smaller, more localized conformational change. Here, the Polλxt-R-SD2β mutant fails to transition to an open state, likely hindered by the permanently closed conformation of Polλ’s catalytic domain [34]. As previously discussed, in Ptet-PolXs, the induced-fit mechanism is limited to a local side-chain movement exchanging salt-bridge partners within the active site, without the need for a complete opening of the active site during the catalytic cycle. Instead, the hybridization of a correct nucleotide with DNA might trigger its optimal positioning, facilitating the release of the catalytic aspartate from its interaction with K534. However, although Ptet-PolXs possess a Loop3, it does not seem to play a role in their fidelity, at least in the gap-filling activity with a continuous template strand, perhaps due to its lack or loss of functionality. Ultimately, Ptet-PolXs exhibit sequence features of both Polβ and Polλ but only one fidelity mechanism partially resembling that of Polβ. From an evolutionary perspective, this could suggest that Ptet-PolXs represent a common ancestor of both metazoan Polβ and Polλ (Fig. 8A).

### Conclusion and perspectives

In summary, our study reveals that PolXs of *P. tetraurelia* exhibit distinct behaviors depending on the DNA substrate and that they display features of both Polλ and Polβ. Specifically, we propose that PolXa is specialized in gap filling in a DSB context, while PolXc and PolXd are more efficient in gap filling in a single-strand break context. In contexts involving gap filling with a non-continuous but complete template strand, all Ptet-PolXs exhibit high specificity.

The *in vitro* data presented here complement *in vivo* findings from Verron *et al.* [15], who investigated the BRCT and linker domains of PolXa and PolXc. Their results showed that PolXa specifically contributes to DNA rearrangements through overexpression and specific recruitment by NHEJ factors such as Ku70a/80c through its linker domain. We extend these observations by showing that PolXa’s catalytic core exhibits superior activity in repairing DSBs induced by PGR. We also propose that when both strands need to be filled by DNA polymerases, the other Ptet-PolXs could participate in secondary DSB repair through two pathways (Fig. 7).

Our study also suggests a fidelity mechanism in Ptet-PolXs, triggered by the entry of a correct dNTP and DNA in the active site, akin to a local induced-fit activation. Although this mechanism is supported by indirect structural evidence, functional assays clearly indicate that residues involved in this putative induced-fit mechanism play a role in fidelity. We also find that Loop3 is important for Polλ’s fidelity but not in Ptet-PolXs, in a single-nucleotide gap-filling context.

Surprisingly, other DNA polymerases of family X also share residues central to the two fidelity mechanisms: Plants PolXs (cluster #4 in Fig. 2) display a lysine equivalent of Polβ’s R258 and also contain a Loop3, similar to HsPolλ and Ptet-PolXs. Their consensus steric gate (AWTGN) and SD2 (DDT) motifs also diverge from known sequences, suggesting that further studies could uncover a similar function in DNA repair.

From an evolutionary perspective, *P. tetraurelia* PolXs may represent a missing link between Polβ and Polλ. Our CLANS analysis of the entire PolX family can help to refine the evolutionary scenario proposed by Bienstock *et al.* [65], who suggested that the PolX family originated from bacterial PolX, followed by yeast PolIV, which then separated in eukaryotes into two groups: Polμ/TdT and Polλ/Polβ. We propose that the Polμ/TdT group could originate from the Polμ-like group of PolX found in fungi, while eukaryotic Polλ and Polβ could originate from the fungal Polλ-like group through PolX found in plants and ciliates like *P. tetraurelia*, as these groups display similarities and share traits of both Polλ and Polβ fidelity mechanisms (Fig. 8A). This hypothetical scenario would be in line with the place of *Paramecium* in the life tree (Supplementary Fig. S3, based on [76]).

Finally, the role and structure of the conserved linker domain in *Paramecium* PolXs is intriguing. This domain, which differs significantly from human Polλ, could play a key role in the dynamics of the NHEJ complex in *Paramecium*. Disorder and AlphaFold predictions suggest that this domain may contain secondary structure elements such as α-helices (Supplementary Fig. S1). Its structured nature could enhance the precise positioning of the NHEJ complex and DNA, optimizing repair efficiency. Such a structured linker domain could be essential in *Paramecium*’s specialized NHEJ system, enabling the precise and efficient positioning of a DNA substrate of a unique type, and the attached DNA polymerase during DNA end processing. This is feasible because there is only a single type of DNA substrate to manage. In contrast, metazoan Polλ and Polμ must deal with a wide variety of DNA ends (e.g. varying microhomology lengths, different orientations of microhomologies, …) resulting from various DNA DSBs. For *Paramecium* PolXa, the ability to stabilize its unique physiological substrate in coordination with the NHEJ machinery would be highly advantageous. The linker domain’s sequence variability between the four Ptet-PolXs (56.5% of identity between PolXa and PolXc) suggests that it may also contribute to the specialization of PolXa, as previously proposed by Verron *et al.* [15]. Further structural characterization of the full NHEJ short-range complex in the presence of PolX is needed to explore this hypothesis. We note that plant DNA PolX also has a long linker domain, which is not SP-rich, but is predicted to be disordered by PONDR and AlphaFold3. It would be interesting to check whether it contributes to the co-localization of the plant PolX with the rest of the NHEJ machinery that is at work, in particular, during DNA DSB repair after the action of CRISPR-Casp genetic engineering [41].

## Supplementary Material

gkaf786_Supplemental_File

## Data Availability

The crystallographic data generated in this study have been deposited to the PDB under the codes 9EWB, 9EWC, 9EWD, 9EWE, and 9EWG.
