# Correction to ‘Fidelity, specialization, and evolution of *Paramecium* PolX DNA polymerases involved in programmed double-strand break DNA repair’

**DOI:** 10.1093/nar/gkaf1163

**Published:** 2025-10-30

This is a correction to: re Antonin Nourisson, Sophia Missoury, Soizick Lucas-Staat, Ahmed Haouz, Marc Delarue, Fidelity, specialization, and evolution of *Paramecium* PolX DNA polymerases involved in programmed double-strand break DNA repair, *Nucleic Acids Research*, Volume 53, Issue 15, 28 August 2025, https://doi.org/10.1093/nar/gkaf786

In the originally published version of the manuscript the names of the authors were transposed. These have now been order emended in the article.

