# Supplementary material for: Fidelity, specialization, and evolution of Paramecium PolX DNA polymerases involved in programmed double-strand break DNA repair
Source: Nucleic Acids Res. 2025 Aug 19;53(15):gkaf786. doi: 10.1093/nar/gkaf786 (PMC12362244; doi:10.1093/nar/gkaf786)
Supplement: gkaf786_Supplemental_File [file gkaf786_supplemental_file.pdf]

Supplementary material

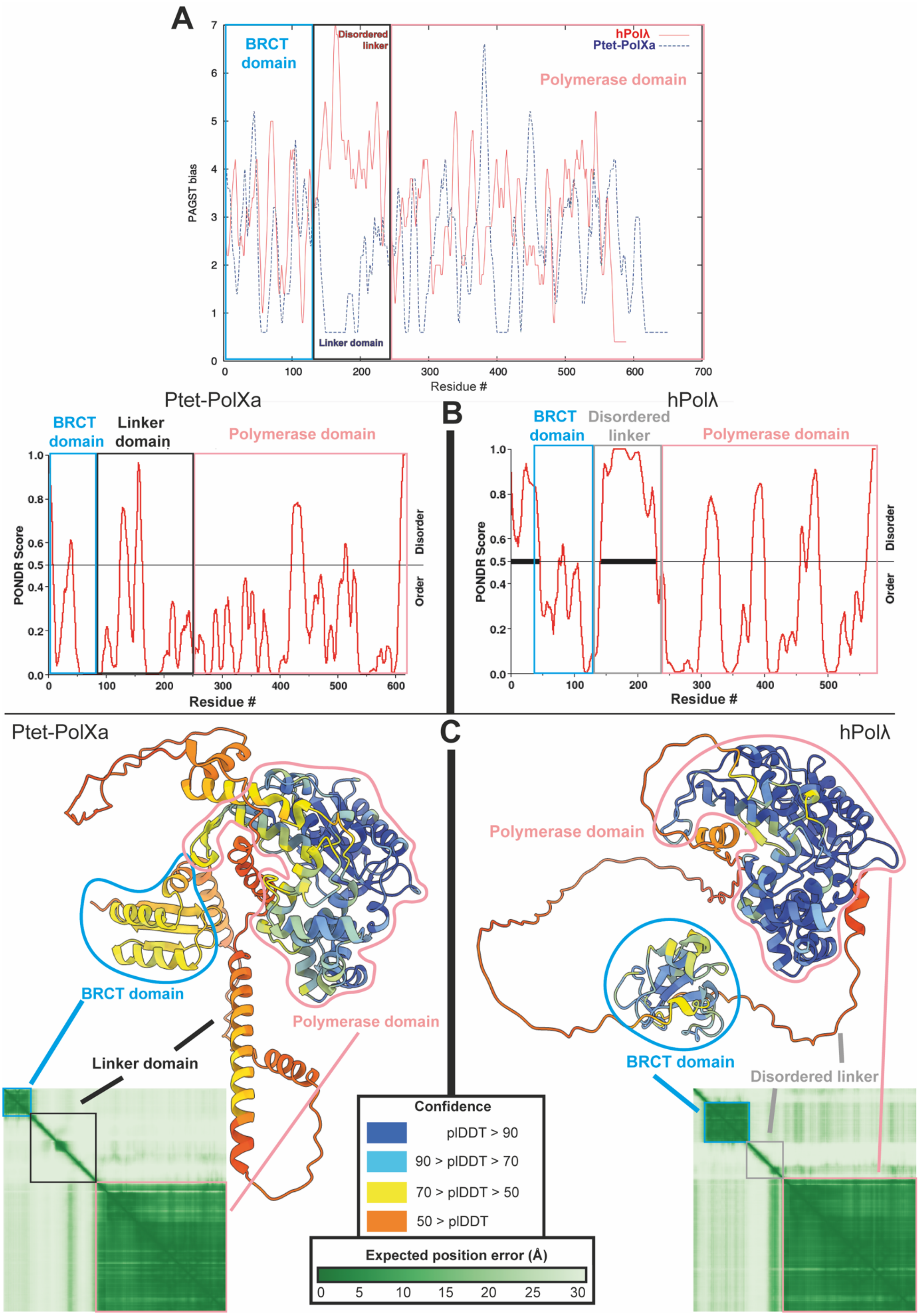

Figure S1 AlphaFold and other bioinformatics predictions of *Paramecium* PolXs structure.

A: PAGST bias score of PtetPolX vs human Polλ, as a function of residue number. A striking difference is seen in the linker domain, where Polλ has an excess of small and polar residues (PAGST) whereas PtetPolX have a shortage of such residues.

B: PONDR (47) predicts a clear disordered region in the linker domain for Polλ, but not for PtetPolX.

C: AlphaFold3 predicts a number of α-helices in the linker domain of PtetPolX, which anchor relatively clearly the BRCT domain to the polymerase domain, whereas there is no way to predict the orientation of the BRCT domain for human Polλ, with respect to the polymerase domain.

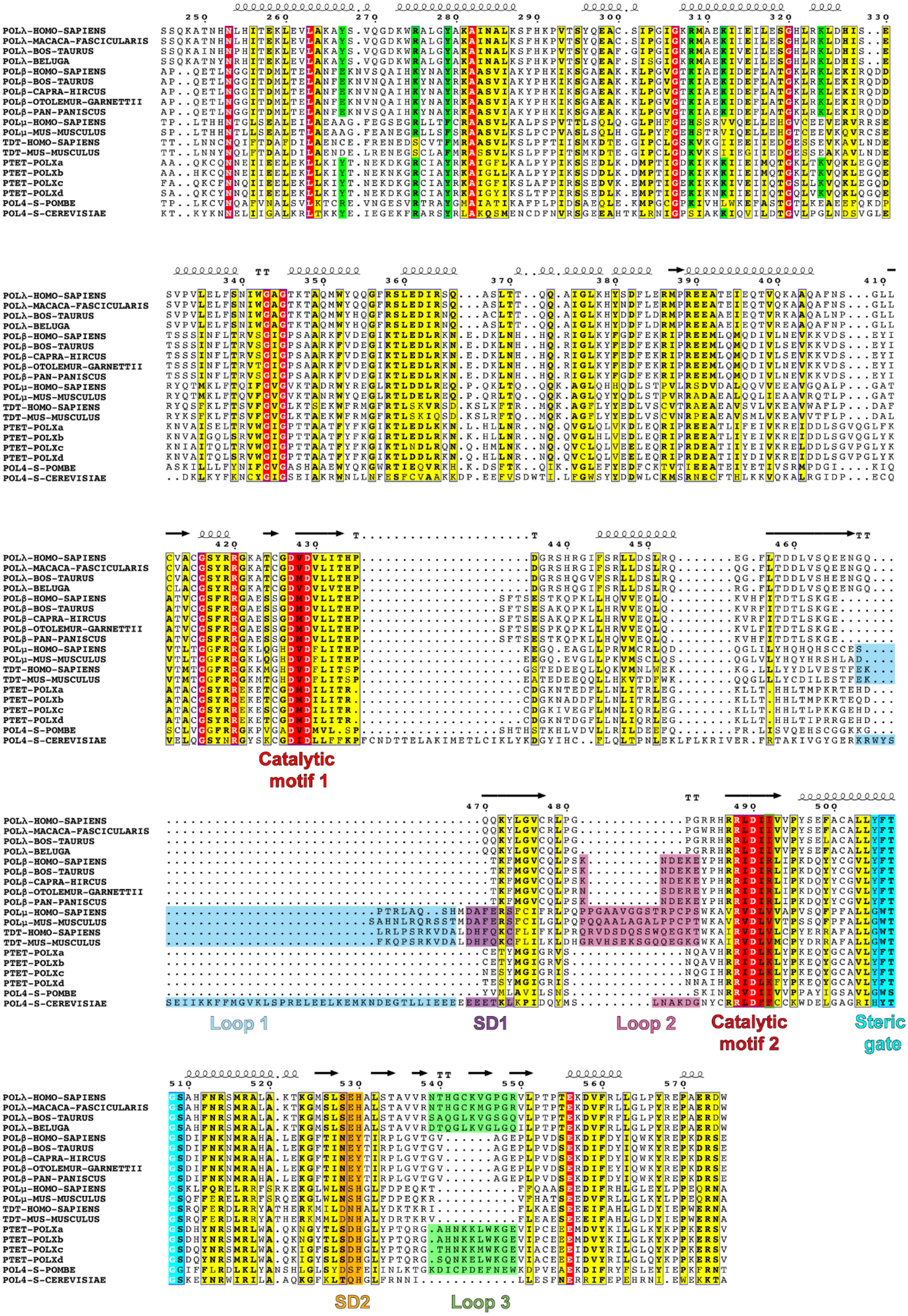

Figure S2 Multiple sequence alignment of the polymerase domains from *Paramecium* PolXs (Ptet-PolXa, b, c, and d) with metazoan DNA polymerase λ, β, μ, and TdT, as well as yeast DNA polymerase IV or Pol4

sequences. Conserved residues implicated in dRP lyase activity and/or 5'P recognition are highlighted in green. Catalytic motifs DxD and RxDx(ϕ/+) are highlighted in red. Loop1 is depicted in blue. The SD1 motif is shown in magenta, Loop 2 is in light pink, and the SD2 is displayed in orange (identified in Gouge *et al.*, EMBO J 2015). The steric gate motif is represented in cyan. Loop3 is indicated in light green.

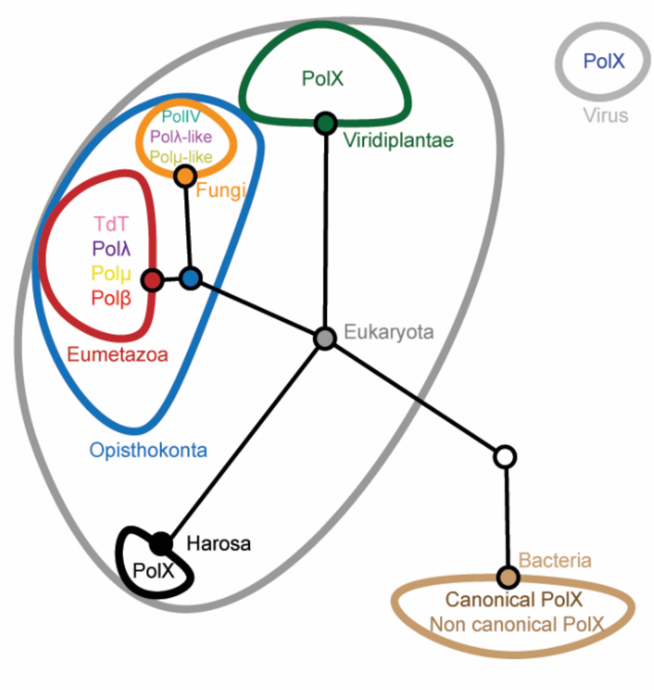

Figure S3 A possible life tree of the 12 groups of PolXs as suggested by CLANS clustering. Ptet-PolXs are in the Harosa group (cluster #9, in black), also called SAR (Stramenopiles, Alveolatas, Rhizaria) or TSAR (if Telonemia are included). Colored circles indicate major domains of life (Eukaryota in grey, Bacteria in brown, Virus in light grey), clades (Opisthokonta in blue, Harosa in black), and kingdoms (Eumetazoa in red, Fungi in orange, Viridiplantae in green). Tree structure based on Lifemap (https://lifemap.cnrs.fr/).

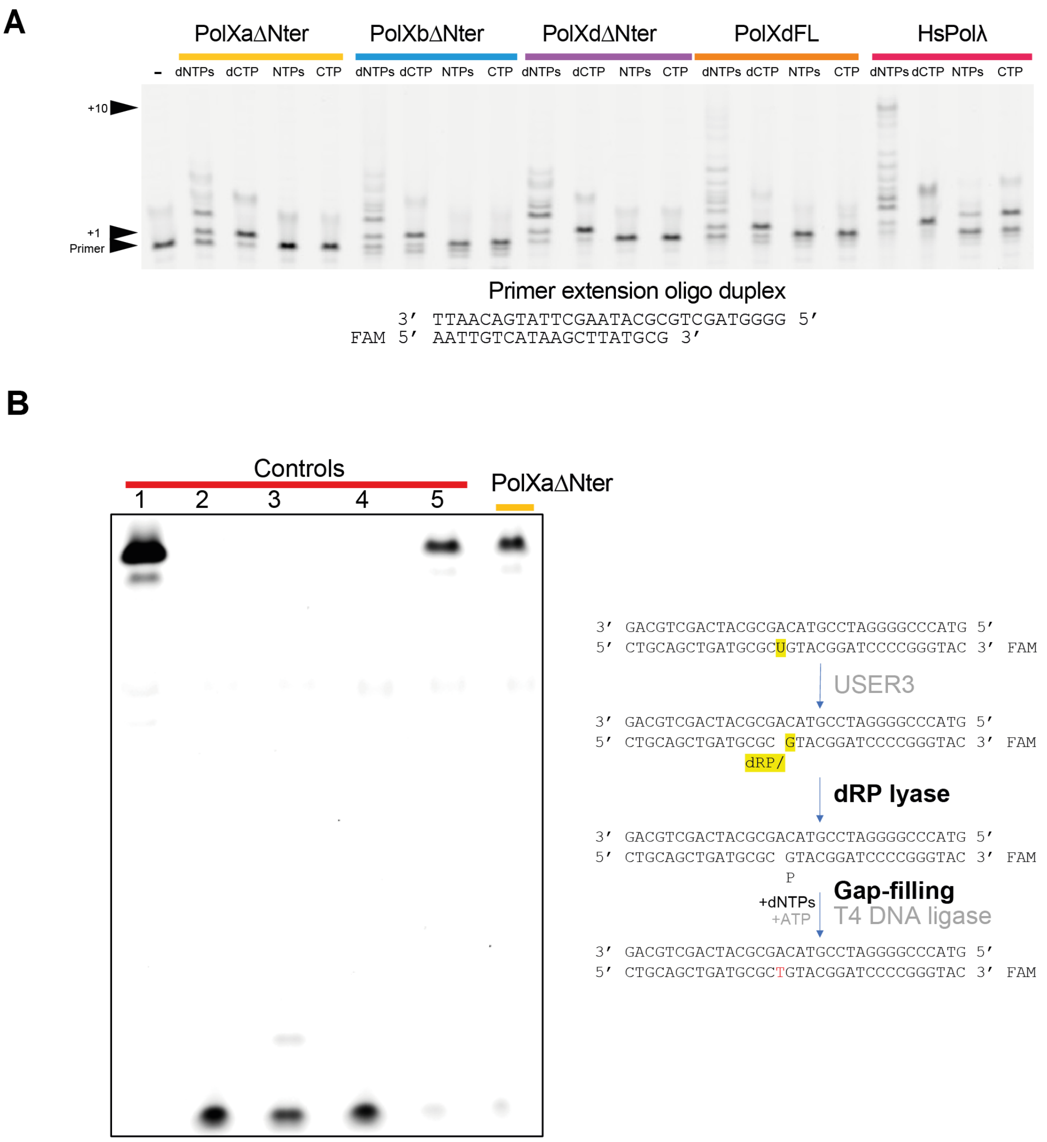

Figure S4 Primer extension activity and dRP lyase assays of *Paramecium* PolX.

A: *Paramecium* PolXs can extend a primer, although not very efficiently. Primer extension assay were conducted at 1 µM of *Paramecium* PolXs and human Polλ in presence of either dNTPs, dCTP only, NTPs or CTP only.

B: PolXaΔNter displays a dRP lyase activity like the one of human Polβ. The schematic on the right illustrates the principle of this assay. Five control lanes are shown on the gel: 1) DNA prior to USER3 treatment (Full-length with dU). 2) DNA post-USER3 treatment without DNA polymerase or DNA ligase. 3) DNA following USER3 treatment and addition of PolXaΔBRCT only. 4) DNA following USER3 treatment and addition of T4 DNA ligase only. 5) Positive control featuring human DNA polymerase β.

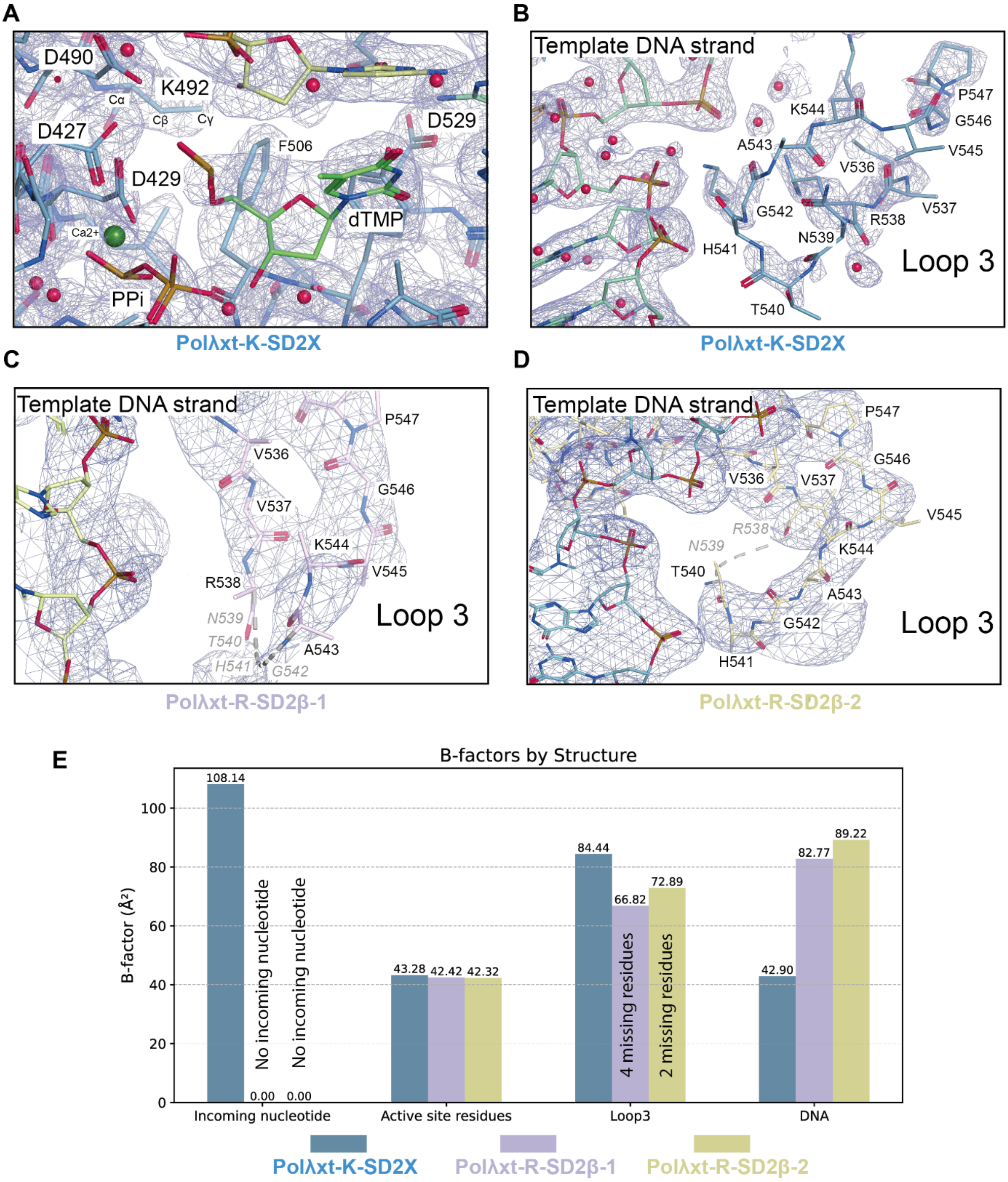

Figure S5 Electron density of the characteristic regions of the studied mutants and their B-factors.

For the studied mutants, the active site (panel A) or Loop3 and DNA (panels B, C, and D) are shown in ball-and-stick representation with their corresponding electron density (2mFo–DFc maps contoured at 1σ). The residues discussed in this study are highlighted and labeled. Calcium ions are represented as green spheres, and water molecules as small red spheres. Panel E shows the mean B factors (in Å^2) for each mutant structure, focusing on the incoming nucleotide, active site residues (D427, D429, D490, Y505, F506, residue 492, and SD2 motif residues), Loop3 residues, and DNA.

Supplementary table S1 Codon optimized nucleotide sequences of the constructs used in this study.

Construct	Nucleotide sequence (codon optimized)
PolXaΔNter	AAACAGCAGTTTTGGGAAGCAAAGAAAGGTTATTTTGTTTGTGAAGCCGGTGCAGCACAGAAATGTCAGAATAA<br>CGAAATTATCGAAGAACTGGAAAAGCTGCTGAAGATCTATACCAACGAAAAAGATAAAGGTCGCTGCATTGCA<br>TATCGTAAAGCAATTGGTTTTCTGAAAGCACTGCCGTATCCGATTAAAAGTAGCGAAGATCTGAAAGATATGCC<br>GACCATTGGTGACAAAATCAAAAAGAAAATCATCGAAATCATGCAGACCGGCAAACTGACCAAAGTTCAGAAA<br>CTGGAAGGTCAAGAGAAAAATGTTGCAATTAGCGAACTGACCCGTGTTTGGGGTATTGGTCCGACCACCGCAG<br>CAACCTTTTATTTCAAAGGTATTAAGACCCTTGAGGACCTGAAAAAGAATCTGCATCTGCTGAATCGTAATCAGC<br>AGGTTGGTCTGCAGCTGGTTAAAGATCTGGAACAGCGTATTCCGCGTGAAGAAGCAACCCTGATTTTTGAAATT<br>GTGAAACGCGAAATCGATGATCTGAGCGGTGTTCAGGGTCTGTTTAAAGCAACCGCATGTGGTAGCTATCGTCG<br>CGAAAAAGAAACCTGTGGTGATATGGATATTCTGATTACCCGTTGTGATGGCAAAAACACCGAAGATTTTCTGC<br>TGAACCTGATTACACGTCTGGAAGGTAAACTGCTGACCCATCATCTGACCATGCCGAAACGTACCGAACATGAT<br>TGCGAAACCTATATGGGTATTGGCCGTGTTAGCAATCAGGCAGTTCATCGTCGTATTGATCTGAAACTGTATCCG<br>AAAGAACAGTATGGTTGTGCCGTTCTGTATTTTACCGGTAGCGATCATTATAATCGTAGCATGCGTCTGTGGGCA<br>CAGAAAAATGGTTATACCCTGAGCGATCATGGTCTGTATCCTACACAGCGTGGTGCACATAACAAAAAGCTGTG<br>GAAAGGTGAAGTTATTCCGTGCGAAGAAGAAATGGACGTCTATAAAATCCTGGGCCTGAAATACAAACCGCCT<br>AAAGAACGTAGCGTGTAA
PolXbFL	ATGTTCAAAGATATCAGCTTTATGTTTCCGCTGAGCACCGTTAATCTGACCAATACACGTATTCGTACCCTGAGC<br>AGCCTGATTGAAAGCAATGGTGGTGAAATGCGTCTGAGTCCGGATGCACTGATGATTGTTGGTAGTGATGCAAC<br>CGTTGAACTGTGTCAGAAACAGTTTATCAAATTTAACCTGGATTTCGAACAGTATCGCTACCAGTTTCTGAATGC<br>AGATTGGGTTAGCCAGAGCCTGCTGCATAAAAAACTGCAGCAGAAAAAAAAGTTTCAGCTGTTTCCGCAGGTTG<br>AATTTCAGCCGGTTCTGGAAGAAGTGCCGGAACTGACCACCAAACTGGTTTATGTTGAAGCACTGGCAAAAACC<br>GTTCCGGTTCGTGATGAAATGACCCAGCAGGATGATGGTGATACCCATGTGTATAACATCAAAGATGTTGAACT<br>GCGCACCAACTATGAAAAACGCATGGAAGAATTTCGTCGCGATATCGTGAAAGAATATCGTCAGATTCTGGATT<br>ACGAGTATGATTATGATCTGGACAACTTCCACAACCAGATTGATGATGGTTATGAGTATCTGCTGGAAGATCTGA<br>GCATCCTGAAAAAAGAAGAACCGCAGATCCTGGAAAGCGAGAAATTCTATGGTGATGATGATTGCCTGATTAC<br>CGAAACACGTAAAGTGCAGTTTGATATTCTGGATGGTCTGGATCTGGGTAAAAGCGTTGTTAATGTTGATCAGCC<br>GATTCTGAGTGCCCAGCTGAAAGAAACCAAACAGTTCAATCCGGGTCTGAAACGTAAACAGCAGTTTTGGGAA<br>GCCAAAAAAGGTTATTTTGTTTGTGAAGCCGGTGCAGCACATAAATGCCAGAATAACGAAATTATCGAAGAACT<br>GGAAAAGCTGCTGAAAATCTATACCAACGAGAAAAACAAAGGTCGCTGTATTGCATATCGTAAAGCAATTGGTC<br>TGCTGAAAGCACTGCCGTATCCGATTAAAAGCAGTGATGATCTGAAAGATATGCCGACCATTGGCGACAAAATC<br>AAAAAAAAGATCATCGAAATTATGCAGACCGGCAAACTGACCAAAGTTCAAAAACTGGAAGGCCAAGAAAAA<br>AATGTTGCCATTGGTCAGCTGAGCCGTGTTTGGGGTATTGGTCCGACCACCGCAGCAACCTTTTATTTCAAAGGC<br>ATTCGCACCCTGGAAGATTTGCGTAAAAACAAACATCTGCTGAATCGCAATCAGCAGGTTGGCCTGCATCTGGT<br>TGAAGATCTGGAACAGCGTATTCCGCGTGAAGAGGCAACCCTGATTTATGAAATTGTGAAACGCGAAATTGATG<br>ACCTGAGCGGTGTTCAGGGTCTGTATAAAGCAACCGCATGTGGTAGCTATCGTCGTGAAAAAGAAACCTGTGGT<br>GATATGGATATTCTGATTACCCGTTGCGACGGTAAAAATGCAGATGATTTTCTGCTTAATCTGATCACCCGTCTG<br>GAAGGCAAACTGCTGACCCATCATCTGACCATGCCGAAACGTACCGAACAGGATTGCGAAACCTATATGGGTA

	TTGGCCGTGTTAGCAATCAGGCAGTTCATCGTCGTATCGATCTGAAACTGTATCCGAAAGAACAGTATGGTTGT<br>GCCGTTCTGTATTTTACCGGTAGCGATCATTATAATCGTAGCATGCGTCTGTGGGCACAGAAAAATGGTTATACC<br>CTGAGTGATCATGGTCTGTATCCTACACAGCGTGGTGCACATAACAAAAAACTGTGGAAAGGTGAAGTTATCCC<br>GTGCGAAGAAGAAATGGACGTTTACAAAATTCTGGGCCTGAAATACAAACCGCCTAAAGAACGTAGCGTGTAA
PolXbΔNter	AAACAGCAGTTTTGGGAAGCCAAAAAAGGTTATTTTGTTTGTGAAGCCGGTGCAGCACATAAATGCCAGAATAA<br>CGAAATTATCGAAGAACTGGAAAAGCTGCTGAAGATCTATACCAACGAAAAAAACAAAGGTCGCTGCATTGCA<br>TATCGTAAAGCAATTGGTCTGCTGAAAGCACTGCCGTATCCGATTAAAAGCAGTGATGATCTGAAAGATATGCC<br>GACCATTGGCGACAAAATCAAAAAAAAGATCATCGAAATTATGCAGACCGGCAAACTGACCAAAGTTCAGAAA<br>CTGGAAGGCCAAGAAAAAAATGTTGCCATTGGTCAGCTGAGCCGTGTTTGGGGTATTGGTCCGACCACCGCAG<br>CAACCTTTTATTTCAAAGGTATTCGTACCCTGGAAGATCTGCGCAAAAACAAACATCTGCTGAATCGTAATCAGC<br>AGGTTGGTCTGCATCTGGTTGAAGATCTGGAACAGCGTATTCCGCGTGAAGAGGCAACCCTGATTTATGAAATT<br>GTGAAACGCGAAATTGATGACCTGAGCGGTGTTCAGGGTCTGTATAAAGCAACCGCATGTGGTAGCTATCGTCG<br>TGAAAAAGAAACCTGTGGTGATATGGATATTCTGATTACCCGTTGTGATGGCAAAAACGCAGATGATTTTCTGCT<br>GAACCTGATTACACGTCTGGAAGGTAAACTGCTGACCCATCATCTGACCATGCCGAAACGTACCGAACAGGATT<br>GCGAAACCTATATGGGTATTGGCCGTGTTAGCAATCAGGCAGTTCATCGTCGTATCGATCTGAAACTGTATCCG<br>AAAGAACAGTATGGTTGTGCCGTTCTGTATTTTACCGGTAGCGATCATTATAATCGTAGCATGCGTCTGTGGGCA<br>CAGAAAAATGGTTATACCCTGAGCGATCATGGTCTGTATCCTACACAGCGTGGTGCACATAACAAAAAACTGTG<br>GAAAGGTGAAGTTATCCCGTGCGAAGAAGAAATGGACGTTTACAAAATTCTGGGCCTGAAATACAAACCGCCT<br>AAAGAACGTAGCGTGTAA
PolXcΔNter	ATGCCGACCATTGGCGAAAAGATCAAAAAGAAGATCGTGGAAATCATCCAAACCGGCAGCTTGCTCAAGGTGC<br>AAAAATTGGAAGGTCAGGACAAAAACATTGCGATTACCCAGCTGACCCGTGTTTGGGGCATCGGTCCAACCAC<br>GGCTGCTACCTTTTATTTCAAAGGTATCAAGACCTTAAACGACCTGCGTCAGAATCAGCATATGTTGAATAAGAA<br>CCAACAGGTTTGTCTGCAACTGGTCGAAGAGCTGGAGCAGCGCATCCCGCGTGATGAAGCGACCATTATCTATG<br>AAATTGTTAAACGCGAACTGGATGATCTCTCGGGTGTACCGGGTCTATACAAGGCGACTGCCTGCGGTTCGTAC<br>CGTCGCGAAAAGGAGTCTTGTGGTGATATGGATATTCTGATCACGCGTTGTGACGGTAAGATCGTGGAGGGTTT<br>CCTGATGAATCTGATACAGCGCCTGGAAGGCAAACTGCTCACGCATCACCTGACTCTGCCGAAGAAAGGTGAG<br>CACGACAACGAGAGCTATATGGGTATTGGCCGCATCAGCAATCAAGGTATTCATCGTAGAATTGACCTGAAACT<br>GTACCCGAAGGAGCAATATGGTTGCGCAGTTCTGTACTTCACCGGCAGTGACCAGTACAACCGTTCCATGCGTT<br>TATGGGCACAGAAAATCGGCTTCTCCCTGAGCGATCATGGCTTGTACCCGACGCAGCGTGGTACACACAACAA<br>AAAGATGTGGAAAGGGGAGGTGATTGCGTGCGAAGAAGAGATCGACGTCTATCGCATTCTGGGCCTGCAGTAT<br>AAACCACCGAAAGAGCGGTCCGTGTAA
PolXdΔNter	CAGATTCAGTATTGGGAAAACAAACGCGAATTTTTCATTTGTGATGCAGGTAGCGCACAGAAATGCTATAACAA<br>CCAGATTATCGAGGAACTGGAAAAGCTGCTGAAAATCTATACCAACGAGAAAGATAAAGGTCGCTGTATTGCAT<br>ATCGTAAAGCCATCGGTTATATCAAGAGCCTGACCTTTCCGATTCGTAGCAGCGAAGATCTGAAAGAAATGCCG<br>ACCATTGGCGAGAAAATCAAGAACAAAATCATCGAGATTATCCAGACCGGTCAGCTGGTTAAAGTTCAGAAACT<br>GCAGGGTCAAGAAAAAAATGTTGCAATTACCCAGCTGAGCCGTGTTTGGGGTATTGGTCCGACCACCGCAGCA<br>ACCTTTTATTTCAAAGGTATTAAAACCCTGGACGACCTGCGTAAAAATCAGCATCTGCTGAATCGTAATCAGCAG<br>GTTTGTCTGCAGCTGGTTGAAGAACTGGAACAGCGTATTCCGCGTGATGAAGCAACCATTATCTATGATATTGTG<br>AAACGCGAGATCGATGATCTGAGCGGTGTTCCGGGTCTGTATAAAGCAACCGCATGTGGTAGCTATCGTCGTGA<br>AAAAGAAACCTGTGGTGATATGGATATTCTGATTACCCGTTGTGATGGCAAAAACACCGATGGTTTTCTGCTGAA<br>CCTGATTCAGCGTCTGGAAGGTAAACTGCTGACCCATCATCTGACCATTCCGCGTCGTGGTGAACATGATACCG

	AAAGCTATATGGGTATTGGCCGTATTAGCAATAATGCCATTCATCGTCGTATCGACCTGAAATTCTATCCGAAAG<br>AACAGTATGGTTGTGCCGTTCTGTATTTTACCGGTAGCGATCAGTATAATCGTAGCATGCGTCTGTGGGCACAGA<br>AAATTGGTTATAGCCTGAGCGATCATGGTCTGTATCCGACACAGCGTGGTAGCCAGAATAAAGAACTGTGGAAA<br>GGTGAAGTTATTGCCTGCGAAGAAGAAATTGACGTTTATCGTATTCTGGGCCTGCAGTATAAACCGCCTAAAGA<br>ACGTAGCGTTTAA
PolXdΔNter-Loop3β	ATGCCGACCATTGGCGAGAAAATCAAGAACAAGATCATCGAGATCATCCAGACCGGTCAACTGGTTAAGGTGC<br>AGAAACTACAGGGCCAAGAAAAAAACGTGGCTATTACCCAGCTTTCCCGTGTTTGGGGCATCGGTCCGACGAC<br>CGCGGCAACGTTCTATTTCAAAGGCATCAAAACTCTGGATGACCTGCGTAAGAACCAACATCTGTTGAACCGTA<br>ATCAGCAGGTTTGTCTCCAGCTGGTAGAAGAGTTGGAGCAGCGTATCCCGCGGGATGAAGCAACTATTATTTAT<br>GACATTGTCAAGCGCGAGATCGACGATCTGAGCGGTGTTCCGGGTTTGTACAAAGCGACTGCGTGTGGTTCCTA<br>CCGCCGTGAGAAGGAGACATGCGGTGACATGGATATTCTGATTACGCGCTGTGACGGCAAGAACACCGATGGT<br>TTTCTGCTGAACCTCATCCAACGTCTGGAAGGTAAGCTTTTAACCCACCACCTGACCATTCCGAGACGTGGTGAG<br>CACGATACCGAAAGCTATATGGGTATTGGTCGTATCAGCAATAATGCGATTCATAGACGTATCGATCTGAAGTTC<br>TACCCGAAAGAGCAATATGGGTGCGCAGTTCTGTACTTCACCGGCAGCGATCAGTACAATCGCAGCATGCGTTT<br>GTGGGCTCAGAAGATAGGCTACAGCTTGTCGGACCATGGTCTGTATCCGACCCAACGCGGTGGTGTGGCGGGT<br>GAGGTGATCGCCTGCGAAGAAGAAATCGACGTGTACCGTATTCTGGGCCTGCAATACAAGCCTCCGAAAGAAC<br>GTAGCGTGTAA
PolXdFL	ATGTTTAACGCCATCAGCTTTATGTTTCCGCTGAGCACCGTTAATCTGACCAATAATCGTATTAAAACCCTGCGC<br>AATCTGATTGAACGTAATGGTGGTACAATTGAACTGAATAGCCGCACCATTATGATTGTTGGTAGTGATGCAACC<br>AGCGAAAGCTGTCAGAAACAGCTGGAAAAAATGCACCTGAACTTTGAACAGTATCGCCAGCAGTTTATTAACGC<br>AGATTGGATTAGCCAGAGTCTGCAGGCAAAAAATCTGCTGGATTTCAAAAAATACCAGCTGTTCAGCGAAATCG<br>AGCAGAAACAGAAACGTGTTAGTCCGCAGAGTACCGATACCAAATTTGTTTATATTGAGAGCCTGGCAAAAACC<br>GTTCCGCTGAAAGAAGATGCCGAAGATAGCCTGGATATGGAAAGCGGTGAATATACCATTATCAAACCGGAAA<br>TGCGCGAGAAATACGAGAAAAAAAAGCAAGAATTCAAACGCCAGATGATCAAAGAAAATCGTTTCCTGCTGGA<br>CTACGAGTATGATAAAGATCTGGATAACTATCACCACCAGGTTGATGATGGTTATGAATTTCTGCTGGACAACTT<br>CCAGATCCTGAAAAAAGAAGAGTTCGACGCACCGCAGCAGAATAAATTCTATGGTGATGAAGATTGCCAGATC<br>ACCGAAATTCGTAAACCGAGCATTGATATTTTTCAAGGTCTGGACATGGGTAAAGCCCTGGTTAATGTTGATCAG<br>CCGCTGGTGAATGCACAGATTAAAGAAACCAAACAGTTTCAGCCTGGTAGCAAACAGCAGATTCAGTATTGGG<br>AAAACAAACGCGAATTTTTCATTTGTGATGCAGGTAGCGCACAGAAATGCTATAACAACCAGATTATCGAGGAA<br>CTGGAAAAGCTGCTGAAAATCTATACCAACGAGAAAGATAAAGGTCGCTGTATTGCATATCGTAAAGCCATCGG<br>TTATATCAAGAGCCTGACCTTTCCGATTCGTAGCAGCGAAGATTTAAAAGAAATGCCTACCATCGGCGAGAAGA<br>TCAAAAACAAAATCATCGAAATCATCCAGACCGGTCAGCTGGTTAAAGTTCAGAAACTGCAGGGTCAAGAAAA<br>AAATGTTGCAATTACCCAGCTGAGCCGTGTTTGGGGTATTGGTCCGACCACAGCAGCAACCTTTTATTTCAAAGG<br>CATTAAGACCCTGGATGACCTGCGTAAAAATCAGCATCTGCTGAATCGTAATCAGCAGGTTTGTCTGCAGCTGG<br>TTGAAGAACTGGAACAGCGTATTCCGCGTGATGAAGCAACCATTATCTATGATATTGTGAAACGCGAGATCGAT<br>GATCTGAGCGGTGTTCCGGGTCTGTATAAAGCAACCGCATGTGGTAGCTATCGTCGTGAAAAAGAAACCTGCG<br>GTGATATGGATATTCTGATTACCCGTTGTGATGGCAAAAATACCGATGGTTTTCTGCTGAACCTGATTCAGCGTC<br>TGGAAGGCAAACTGCTGACCCATCATCTGACCATTCCGCGTCGTGGTGAACATGATACCGAAAGCTATATGGGT<br>ATTGGCCGTATTAGCAATAATGCCATTCATCGTCGTATCGACCTGAAATTCTATCCGAAAGAACAGTATGGTTGT<br>GCCGTTCTGTATTTTACCGGTAGCGATCAGTATAATCGTAGCATGCGTCTGTGGGCACAGAAAATTGGTTATAGC

	CTGAGCGATCATGGTCTGTATCCGACACAGCGTGGTAGCCAGAATAAAGAACTGTGGAAAGGTGAAGTTATTG<br>CCTGCGAAGAAGAAATTGACGTTTATCGTATTCTGGGCCTGCAGTATAAACCGCCTAAAGAACGTAGCGTTTAA
HsPolλ	GATCCGCGTGGTATTCTGAAAGCATTTCCGAAACGTCAGAAAATTCATGCAGATGCAAGCAGCAAAGTTCTGGC<br>AAAAATTCCGCGTCGCGAAGAAGGTGAAGAGGCCGAAGAATGGCTGAGCAGCCTGCGTGCACATGTTGTTCGT<br>ACCGGTATTGGTCGTGCACGTGCCGAACTGTTTGAAAAACAAATTGTTCAGCATGGTGGTCAGCTGTGTCCGGC<br>ACAAGGTCCGGGTGTTACCCATATTGTTGTTGATGAAGGTATGGATTATGAACGTGCACTGCGTCTGCTGCGCCT<br>GCCGCAGTTACCGCCTGGTGCACAGCTGGTTAAAAGCGCCTGGCTGAGCCTGTGTCTGCAAGAACGTCGTCTG<br>GTTGATGTTGCAGGTTTTAGCATTTTTATCCCGAGCCGTTATCTGGATCATCCGCAGCCGAGCAAAGCAGAACA<br>GGATGCCAGCATTCCTCCGGGTACACATGAAGCACTGCTGCAGACCGCACTGAGTCCGCCTCCGCCTCCTACAC<br>GTCCGGTTAGCCCTCCGCAGAAAGCAAAAGAAGCACCGAATACGCAGGCACAGCCGATTAGTGATGATGAAG<br>CAAGTGATGGTGAAGAAACCCAGGTTAGCGCAGCCGATCTGGAAGCACTGATTAGCGGTCATTATCCGACCAG<br>CCTGGAAGGTGATTGTGAACCGAGTCCGGCACCGGCAGTTCTGGATAAATGGGTTTGTGCACAGCCGTCAAGC<br>CAGAAAGCCACCAATCATAATCTGCATATTACCGAGAAACTGGAAGTGCTGGCCAAAGCATATAGCGTTCAGG<br>GTGATAAATGGCGTGCACTGGGTTATGCAAAAGCAATTAATGCACTGAAAAGCTTCCATAAACCGGTGACCAGC<br>TATCAAGAAGCCTGTAGCATTCCTGGTATTGGTAAACGTATGGCCGAGAAAATCATTGAAATTCTGGAAAGCGG<br>TCATCTGCGTAAACTGGATCATATTAGCGAAAGCGTTCCGGTTCTGGAACTGTTTAGCAATATTTGGGGTGCAGG<br>CACCAAAACCGCACAGATGTGGTATCAGCAGGGTTTTCGTAGTCTGGAAGATATTCGTAGCCAGGCAAGCCTG<br>ACCACACAGCAGGCAATTGGTCTGAAACATTATAGCGATTTTCTGGAACGTATGCCTCGTGAAGAAGCAACAGA<br>AATTGAACAGACCGTTCAGAAAGCAGCACAGGCATTTAATAGCGGTCTGCTGTGCGTTGCATGTGGTAGCTATC<br>GTCGTGGTAAAGCAACCTGTGGTGATGTTGATGTTCTGATTACCCATCCTGATGGTCGTAGCCATCGTGGTATTT<br>TTAGCCGTCTGCTGGATAGCCTGCGTCAAGAGGGTTTTCTGACCGATGATCTGGTTAGCCAAGAAGAAAACGGT<br>CAGCAGCAGAAATATCTGGGTGTTTGTCGTCTGCCTGGTCCGGGTCGTCGTCATCGTCGCCTGGATATTATTGTT<br>GTGCCGTATAGCGAATTTGCATGTGCACTGCTGTATTTTACCGGTAGCGCACATTTTAATCGTAGCATGCGTGCC<br>CTGGCCAAAACCAAAGGTATGAGCCTGAGCGAACATGCACTGAGCACCGCAGTTGTGCGTAATACCCATGGTT<br>GTAAAGTTGGTCCTGGTCGTGTTCTGCCGACACCGACCGAAAAAGATGTGTTTCGCCTGCTGGGTCTGCCGTAT<br>CGTGAACCGGCAGAACGTGATTGGTAA
HsPolβ	ATGAGCAAACGCAAAGCGCCGCAGGAAACCCTGAACGGCGGCATTACCGATATGCTGACCGAACTGGCGAAC<br>TTTGAAAAAAACGTGAGCCAGGCGATTCATAAATATAACGCGTATCGCAAAGCGGCGAGCGTGATTGCGAAAT<br>ATCCGCATAAAATTAAAAGCGGCGCGGAAGCGAAAAAACTGCCGGGCGTGGGCACCAAAATTGCGGAAAAAA<br>TTGATGAATTTCTGGCGACCGGCAAACTGCGCAAACTGGAAAAAATTCGCCAGGATGATACCAGCAGCAGCAT<br>TAACTTTCTGACCCGCGTGAGCGGCATTGGCCCGAGCGCGGCGCGCAAATTTGTGGATGAAGGCATTAAAACC<br>CTGGAAGATCTGCGCAAAAACGAAGATAAACTGAACCATCATCAGCGCATTGGCCTGAAATATTTTGGCGATTT<br>TGAAAAACGCATTCCGCGCGAAGAAATGCTGCAGATGCAGGATATTGTGCTGAACGAAGTGAAAAAAGTGGAT<br>AGCGAATATATTGCGACCGTGTGCGGCAGCTTTCGCCGCGGCGCGGAAAGCAGCGGCGATATGGATGTGCTGC<br>TGACCCATCCGAGCTTTACCAGCGAAAGCACCAAACAGCCGAAACTGCTGCATCAGGTGGTGGAACAGCTGCA<br>GAAAGTGCATTTTATTACCGATACCCTGAGCAAAGGCGAAACCAAATTTATGGGCGTGTGCCAGCTGCCGAGCA<br>AAAACGATGAAAAAGAATATCCGCATCGCCGCATTGATATTCGCCTGATTCCGAAAGATCAGTATTATTGCGGC<br>GTGCTGTATTTTACCGGCAGCGATATTTTTAACAAAAACATGCGCGCGCATGCGCTGGAAAAAGGCTTTACCATT<br>AACGAATATACCATTCGCCCGCTGGGCGTGACCGGCGTGGCGGGCGAACCGCTGCCGGTGGATAGCGAAAAA<br>GATATTTTTGATTATATTCAGTGGAAATATCGCGAACCGAAAGATCGCAGCGAATAA

Supplementary table S2 Constructs and primers used for directed mutagenesis and generation of the mutants constructs of this study. The mutated constructs used for crystallographic structure determination or enzymatic experiments are indicated in **bold**. The other constructs are intermediate constructs used for generation of other mutants.

Mutated construct	Initial construct	Primers used for mutagenesis
**PolXaΔNter K534A**	**PolXaΔNter**	CGTCGTATTGATCTGGCACTGTATCCGAAAGAACAGTATGG
		ATGAACTGCCTGATTGCTAACACG
**PolXaΔNter K534R**		CGTCGTATTGATCTGAGACTGTATCCGAAAGAACAGTATGG
		ATGAACTGCCTGATTGCTAACACG
**PolXaΔNter-Loop3β**		CGCCACACCACCACGCTGTGTAGGATACAG
		GGTGAAGTTATTCCGTGCGAAGAAGAA
PolλΔNter	**HsPolλ**	GCACAGCCGTCAAGCCAG
		CCCTTGAAAATACAGGTTCTCGCCG
PolλΔNter-Loop1β	PolλΔNter	GAAACCAAATATCTGGGTGTTTGTCGTCTGC
		GCCTTTAACCAGATCATCGGTCAGAAAACCC
**Polλxt** (ΔNter-Loop1β-C544A)	PolλΔNter-Loop1β	ACAACTGCGGTGCTCAGTG
		GCGTAATACCCATGGTGCGAAAGTTGGTCCTGGTCG
**Polλxt-R** (ΔNter-Loop1β-C544A-I492R)	**Polλxt**	CGTCGCCTGGATATTCGTGTTGTGCCGTATAGC
		ATGACGACGACCCGGACCA
**Polλxt-R-SD2β** (ΔNter-Loop1β-C544A-I492R-SD2NEY)	**Polλxt-R**	ATGAGCCTGAACGAATATGCACTGAGCACC
		ACCTTTGGTTTTGGCCAGGG
**Polλxt-K** (ΔNter-Loop1β-C544A-I492K)	**Polλxt**	CGTCGCCTGGATATTAAAGTTGTGCCGTATAGC
		ATGACGACGACCCGGACCA
**Polλxt-K-SD2X** (ΔNter-Loop1β-C544A-I492R-E529D)	**Polλxt-K**	GCCTGAGCGATCATGCACTGA
		TCATACCTTTGGTTTTGGCCAGGG
**Polλ-loop3β** (ΔNter-Loop1β-Loop3β)	PolλΔBRCT-Loop1β	GGTCGTGTTCTGCCGACAC
		CGCCACACCACGCACAACTGCGGTGC
